# Overexpression of MMP Family Members Functions as Prognostic Biomarker for Breast Cancer Patients: A Systematic Review and Meta-Analysis

**DOI:** 10.1371/journal.pone.0135544

**Published:** 2015-08-13

**Authors:** Fanghui Ren, Ruixue Tang, Xin Zhang, Wickramaarachchi Mihiranganee Madushi, Dianzhong Luo, Yiwu Dang, Zuyun Li, Kanglai Wei, Gang Chen

**Affiliations:** Department of Pathology, the First Affiliated Hospital of Guangxi Medical University, Nanning, Guangxi Zhuang autonomous region, China; Wayne State University School of Medicine, UNITED STATES

## Abstract

**Background:**

Matrix metalloproteinases (MMPs) are regarded to be relevant to the prognosis of breast cancer. Numerous studies have confirmed the association between MMPs and tumor growth, invasion and metastasis in breast cancer. However, their prognostic values for survival in patients with breast cancer remain controversial. Hence, a meta-analysis was performed to clarify a more accurate estimation of the role of MMPs on prognosis of breast cancer patients.

**Method:**

A systemic electronic search was conducted in PubMed, Embase and Web of science databases to identify eligible studies, which were associated with the relationship between MMPs and prognosis of breast cancer. The correlation in random-effect model was evaluated by using the hazard ratios (HRs) and 95% confidence intervals (CIs).

**Results:**

A total of 28 studies covering 4944 patients were included for meta-analysis. A summary hazard ratio (HR) of all studies was calculated, as well as the sub-group HRs. The combined HRs calculated by either univariate or multivariate analysis both suggested that overexpression of MMPs had an unfavorable impact on overall survival (OS) (HR = 1.694, 95%CI: 1.347–2.129, P < 0.001; HR = 1.611, 95%CI: 1.419–1.830, P < 0.001, respectively). And the univariate analysis showed that patients with overexpression of MMPs had worse relapse-free survival (RFS) (HR = 1.969, 95%CI: 1.460–2.655, P < 0.001) in all eligible studies. In the sub-group analyses, HRs of MMP-9 positivity with poor OS were 1.794 (95%CI: 1.330–2.420, P < 0.001) and 1.709 (95%CI: 1.157–2.526, P = 0.007) which were separately evaluated by univariate and multivariate analysis. A small number of articles demonstrated that MMP-2 overexpression was not related with shorter OS (HR = 1.400, 95%CI: 0.610–3.029, P = 0.427). Four studies included in the OS analysis of MMPs expression in serum suggested that positive expression of serum MMPs may be an unfavorable factor (HR = 1.630, 95%CI: 1.065–2.494) for breast cancer patients. No publication bias was observed in the current meta-analysis.

**Conclusions:**

Our findings suggested that MMPs overexpression (especially MMP-9, MMP-2, MMPs overexpression in serum) might indicate a higher risk of poor prognosis in breast cancer. Larger prospective studies are further needed to estimate the prognostic values of MMPs overexpression.

## Introduction

Matrix metalloproteinases (MMPs), a family of zinc-dependent endopeptidases, are found in extracellular milieu of various tissues. They are involved in the degradation of extracellular matrix (ECM) [1,2]. To date, 26 MMPs have been known, which share a large amount of common structural and functional similarities, however, differ in their substrate specificities [3]. Based on the specific structure, MMPs not only play a key role in physiological process [4,5], but also account for the cancer invasion and metastasis, angiogenesis and tumorigenesis [6,7].

Breast cancer is the most frequent cancer in women, and its molecular characteristics are the decisive factors related to the behavior of the cancer. In clinical practice, we rely on clinicopathological features to predict tumor behavior and patient outcome. Although early detection and targeted therapies have significantly improved breast cancer-related survival rates, there are still obstacles needed to be overcome.

A number of studies have investigated the association between MMPs expression and survival in breast cancer patients. However, it remains controversial whether MMPs are qualified as prognostic biomarkers or not and no consensus has been reached yet. Scorilas et al.[8], Wu et al.[9] and Bottino et al.[10] reported that the decreased expression of MMP-9 in breast cancer tissues was correlated with poorer prognosis. However, some articles reported independently that breast cancer patients with high MMP-9 expression showed a poor prognosis [11–15]. Besides, a meta-analysis conducted by Song et al.[16] testified that MMP-9 overexpression could act as a biomarker suggesting unfavorable results on both overall survival (OS) (hazard ratio (HR): 1.70, 95% confidence interval (95%CI): 1.41–2.04) and RFS (HR: 1.54, 95%CI: 1.17–2.01) in breast cancer patients. In addition, it also remained conflicting as to the influence of MMP-1, MMP-2, MMP-11, MMP-13 and MMP-14 expression on the survival of breast cancer patients [17–22]. Up to now, there have been no studies identifying the relationship between MMPs family expression and prognostic value in patients with breast cancer. Thus, we performed a meta-analysis of published studies to assess the effects of MMPs family expression in tumor tissue on survival in breast cancer patients.

## Materials and Methods

### Search strategies for identification of studies

Relevant literatures on MMPs expression and survival results in breast cancer patients were searched in PubMed, Embase and Web of science, databases update to January, 2015. The search strategy was based on a combination of Medical Subject Headings (MeSH) and text words i.e. ("Breast cancer" or "Breast carcinoma" or "Breast Neoplasm" or "Breast Tumor") and (“Membrane-Type Matrix Metalloproteinase” or “MMPs” or “Matrix metalloproteinase”) and (“prognostic” or “prognosis” or “survival” or “outcome”). References from identified primary studies and review articles were further searched to find additional eligible studies to avoid missing from electronic searching approaches.

### Criteria and selection process of studies included in this review

Two independent reviewers (FHR and RXT) read the titles and abstracts of all candidate articles. Articles that could not be decided from title and abstract were retrieved for further full-text review. Articles were individually read and checked for inclusion and exclusion. Any disparity in quality assessment and data collection was conversed and reached a final agreement via discussion with the third reviewer (GC).

The following inclusion criteria must be met to ensure the quality of each article: (1) the patients were female and diagnosed as primary breast cancer; (2) MMPs expression was measured in tumor tissue or serum; (3) MMPs protein expression was measured instead of mRNA: (4) the method to evaluate MMPs expression was either immunohistochemistry (IHC) or enzyme linked immunosorbent assay (ELISA); (5) HR and 95% CI could be obtained from the article or calculated based on the information in the paper; (6) articles were in English and mentioned the association of MMPs with overall survival (OS) or relapse free survival (RFS) or disease free survival (DFS) or disease-specific survival (DSS) or progression free survival (PFS); (7) When the same research group published relative articles with the same cohort repetitively, only the most complete and/or latest one was included.

Exclusion criteria for this study were as follows: (1) Reviews, letters to the editors, and articles published in a book or articles not published, (2) articles without OS or DFS or DSS or RFS or PFS or in other languages other than English, (3) articles with only animal experiments.

### Data extraction

The following data extracted from the literatures were included: name of first author, publication time, country, the number of patients, stage of disease, cut-off value, location of MMPs expression in tumor or serum, the percent of MMPs positivity, HR and 95% CI. Although we have tried to contact authors of the original for missing data and enquire about unpublished results, some information above was still not available, which was marked as “not reported (NR)”. Inconsistencies in the data extraction were resolved through debates and consultations.

### Statistical analysis

HR and 95% CI were used to estimate the impact of MMPs expression on survival of breast cancer patients in this meta-analysis. By convention, it implies a worse survival for the group with increased MMPs expression when HR>1. This influence of MMPs expression on survival was considered as statistically significant if the corresponding 95% CI for the pooled HR did not overlap1.When HRs were not clearly reported, it was often possible to calculate from available information, for instance, Kaplan-Meier survive curve could be used to estimate HRs by using the methods according to Tierney et al. [23](2007) and Parmar et al. [24](1998). Kaplan-Meier curves were analyzed by Engauge Digitizer version 4.1 (http://sourceforge.net) which was used to extract the survival rata correlated with the prognosis. HRs could also be calculated if the survival and MMPs status were provided for each case in the study by using SPSS software. Stata version 11.0 was used to carry out the data analyses, while Q-tests and I-squared test were used to estimate the heterogeneity. When inferior or no heterogeneity was present (P ≥ 0.05 or I^2^ ≤ 50%), the random-effects model was used to determine the heterogeneity (P < 0.05 or I^2^ > 50%) in this meta-analysis. If there existed heterogeneity, a sensitivity analysis was performed to find the main studies that might contribute to the heterogeneity. For those meta-analyses containing 10 or more studies, the possibility of publication bias was assessed.

Publication bias was evaluated by the Begg’s funnel plot and Egger’s test. If there was no bias, the graph should appear like a symmetrical inverted funnel. On the contrary, the plot should appear skewed and asymmetrical.

## Results

### Study results

**Fig 1:**
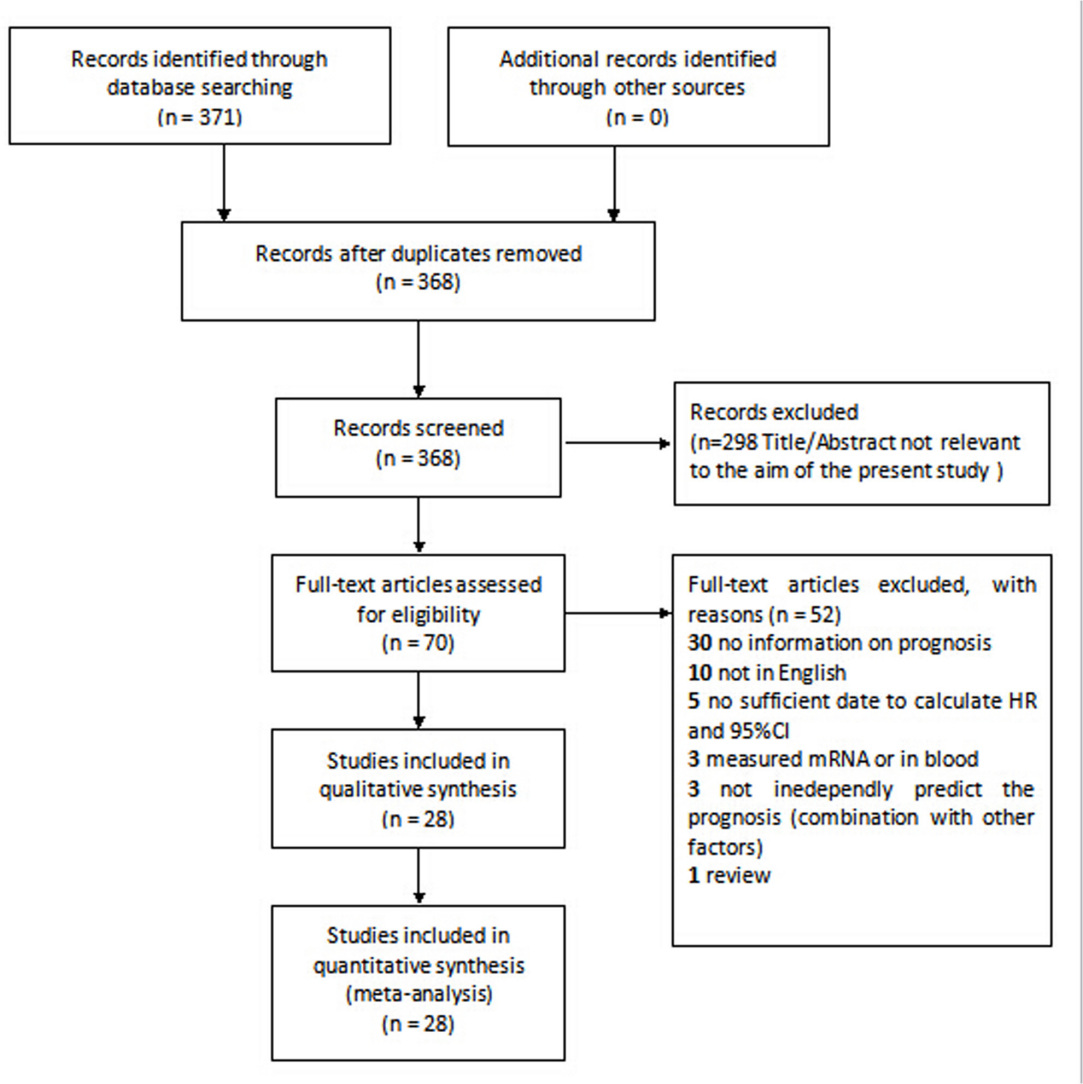
Flow chart of study selection based on the inclusion and exclusion criteria.

The results of the search strategy for studies were summarized in Fig 1, S1 and S2 Files. Finally, 25 (including HR and 95% CI) studies [8–15,17–22,25–35] were eligible for the meta-analysis and three additional studies were included in the systematic review. The main features of the eligible studies for MMPs were summarized in Tables 1 and 2. The total amount of patients included for the current meta-analysis was 5044, ranging from 27 to 453 per study. In total, 18 studies had data on OS with 16 studies of which by univariate analysis and 6 by multivariate analysis. There were 4 studies on DFS and 7 studies on RFS. Moreover, 3 studies and 5 studies had survival date on combination of OS/DFS and OS/RFS, respectively. There also existed 5 studies which observed the expression of MMPs in serum among the included studies. Among the 25 studies, MMP-9 and MMP-2 were estimated in 13 and 8 studies respectively, and two studies evaluated the co-expression of MMP-2 and MMP-9. Immunohistochemistry was the commonest technique to detect MMPs expression, although authors of five articles used ELISA to assess MMPs expression. The cut-off value for definition of MMPs positive expression mainly ranged from more than 0% to 70% and other cut-off values were shown in Table 1. Among all eligible studies for survival analysis, HR values were estimated by the survival data provided in 19 studies and by survival curve in 9 studies. Notably, when HR was given by multivariate analysis instead of univariate analysis, survival curve was applied to calculate another HR as univariate analysis result. Twenty two studies identified MMPs overexpression as an indicator of poor prognosis and another two studies showed the opposite results.

**Table 1:** Main Characteristics of the Eligible Studies in this Meta-analysis.

First Author	Year	Country	Age	N	MMPs(+)	tumor size	Stage (N)	LN status (N)	Location	Method	Cut-Off	follow-up time
Scorilas[8]	2001	Greece	56(median)	210	110(52.4%)	<2cm 48 2~5cm 138	I 24 II 135 III+IV 47	(+)116(-)85	tumor	IHC	NR	62^a^(median)
Wu[9]	2008	China	51	60	serum 38 tumor 46	≤2cm6 >2cm 54	I+II 45 III+IV 15	(+)38 (-)22	serum tumor	ELISAIHC	serum 50ng/ml tumor(++/+++)	>5^b^
Bottino[10]	2014	Brazil	50	27	NR	≤3cm16 >3cm 8	I+II 21 III+I 6	NR	tumor	IHC	190.9au	NR
Mylona[11]	2007	Greece	56.89	175	53(70.3%)	<2cm13 >2cm 40	stromalI 10 II 32 III 11	(+)29 (-)24	tumor stromal	IHC	>20%	95.8^a^
Ranogajec[12]	2012	Croatia	56	138	MMP-2 73(52.9%) MMP-9 117(84.8%)	≤2cm74 >2cm 64	I 21 II 73 III 44	(+)51 (-)60	tumor	IHC	score:2+3+	94^a^
Sung[13]	2012	Korea	46.6	303	NR	<2 cm 159 ≥2cm 144	I 115 II 123 III 65	(+)133 (-)170	serum	ELISA	NR	4.2^b^
Zeng[14]	2013	China	50	253	139(54.9%)	≤3cm 150 >2cm 103	I+II 180 III+IV 73	(+)138 (-)115	tumor	IHC	>0	120–191^a^
Zhao[15]	2013	China	49(median)	127	68(53.5%)	≤2cm59 >2cm 68	I+II 45 III 82	(+)56 (-)71	tumor	IHC	>6-7score	NR
Ahmad[17]	1998	France	55(median)	119	85	<2cm 66 2~5cm49 >5cm 2	NR	(+)74 (-)45	tumor	IHC	NR	60^a^
Sivula[18]	2005	Finland	50(cut-off)	278	161(83%)	>2cm 87	Ductal type only) I 29 II 57 III 61	(+)89 (-)104	tumor	IHC	>20%	10.1^b^
McGowan[19]	2008	Ireland	NR	295	NR	≤2cm155 >2cm140	NR	(+)144 (-)151	tumor	NR	NR	6.7^b^
Zhang[20]	2008	China	50.3	263	12(48.3%)	<2cm46 >2cm 217	I 68 II 119 III 76	(+)135 (-)128	tumor	TMA IHC	SI>6	36–173^a^
Kulic[21]	2012	Croatia	50	60	32(40.5%)	<2cm 39 >2cm 21	I 18 II 20 III 22	(+)21 (-)39	serum	ELISA	4.52 ng/mL	60^a^
Song[22]	2012	Korea	44.5	303	NR	<2 cm 159 ≥2cm 144	I+II 160 III 129	(+)133 (-)170	serum	ELISA	NR	4.24 ^b^
Talvensaari-Mattila[25]	2003	Finland		453	354(78%)	≤5cm 404 >5cm 49	NR	(+)301 (-)152	tumor	IHC	NR	NR
Leppa[26]	2004	Finland	NR	133	65(48.9%)	<2cm61 2~5cm 60 >5cm 9	I 9 II 49 III 32	(+)65 (-)68	serum	ELISA	5.25 ng/ml (median)	5^b^
Li[27]	2004	China	NR	270	mmp-2 153(56.7%) mmp9 161(59.6%) coexpression 124	<2cm 94 2~5cm 156 >5cm 20	I 60 II147 III 63	(+)153 (-)117	tumor	IHC	>1%	61^a^(mean)
Pellikainen[28]	2004	Finland	69.2(mean)	421	217 (52.3%)	<2cm 221 2~5cm166>5cm 24 other 10	I 110 II 192 III 119	(+)148 (-)245	tumor cell stromal cell	IHC	85%	55^a^(mean)
Rahko[29]	2004	Finland	64	168	55%	<2cm72 2~5cm84 >5cm9 others 3	I II III	NR	tumor	NR	50	NR
Bostrom[30]	2011	Finland	57.5	125	67(53.6%)	NR	I 10 II 66 III 49	(+)50 (-)64	tumor	IHC	>70%	20^b^
Sullu[31]	2011	Turkey	52	117	66%	NR	I II III	NR	tumor	NR	CS	NR
Wadowska-Jaszczyńska[32]	2011	Poland	55.2	108	47(35.5%)	NR	I+II 108	NR	tumor	IHC	NR	NR
Fernandez-Guinea[33]	2012	Spain	57(cut-off)	97	49%	<2cm 47 >2cm 50	I 27 II 45 III 25	(+) 51 (-) 46	tumor	IHC	CS	85^a^
Merdad[34]	2014	Arabia	48(mean)	45	NR	3.1(mean)	I 4 II 17 III 17	(+)23 (-)19	tumor	IHC	NR	52.1^a^
Min[35]	2014	Korea	47(median)	177	MMP-2 132(74.6%)MMP-9 166(93.8%)	≤2 cm 63 >2 cm 114	I or II 130 III 47	(+)99 (-)78	tumor/stromal	IHC	IRS>5	NR
Chenard[36]	1996	Ireland	NR	111	NR	<2cm 44 2~5cm 54 >5cm 10	I 15 II 47 III 26	(+)44 (-)43	tumor	IHC	30%	6^b^
Talvensaari-Mattila[41]	2001	Finland	63(median)	100	69(69%)	≤5cm 69 >5cm 10	Ductal infiltratingI 12 II+III 68	(+) all	tumor	IHC	>1%	40.4^a^(median)
Talvensaari-Mattila[42]	1999	Finland	<40 all 41(median)	108	82(76%)	≤5cm 78 >5cm 11	Ductal infiltratingI 4 II+III 100	(+) all	tumor	IHC	>1% (+) >50% ++	57.1^a^(median)

Reported: reported in the article; SC: K-M survival curve; CS: cytoplasmic staining; SI: staining index; ISR: immunoreactive score; NR: not reported

a: month

b: year.

**Table 2:** Survival date on prognosis of the Eligible Studies in this Meta-analysis.

First Author	survival analysis	HR statistics	Univariate HR(95%CI)	Multivariate HR(95%CI)	MMP type
Scorilas[8]	OS,RFS	Reported	OS 0.59(0.33–1.06) RFS 0.65(0.41–1.04)	OS 0.78(0.35–1.75) RFS 0.89(0.49–1.61)	MMP-9
Wu[9]	OS,RFS	Reported	OS 1.22(0.18–8.31) RFS 0.55(0.03–8.82)	NR	MMP-9
Bottino[10]	OS	SC	OS 0.57(0.14–2.3)	NR	MMP-9
Mylona[11]	OS,DFS	Reported SC	NR	OS 2.437(1.271–4.671) DFS 1.842(1.083–3.133)	MT1-MMP MMP-9
Ranogajec[12]	OS	SC	OS MMP-2 13.961(2.619–74.409) MMP-2/9 1.65(0.235–12.5)	NR	MMP-2 MMP-9
Sung[13]	DFS	Reported	NR	DFS 1.02 (0.99–1.06)	MMP-9
Zeng[14]	OS,DFS	Reported	OS 2.288 (1.391–3.763) DFS 2.108(1.364–3.258)	OS 1.993(1.165–3.409) DFS 1.808 (1.125–2.905)	MMP-9
Zhao[15]	OS,PFS	Reported	NR	OS 1.761(1.092–2.840) PFS 1.824(1.122–2.965)	MMP-9
Ahmad[17]	RFS	Reported	RFS 1.96(1.12–3.44)	NR	MMP-11
Sivula[18]	DSS	SC	DSS 0.532(0.164–1.726)	NR	MMP-2
McGowan[19]	OS	Reported	OS 3.65(1.33–9.96)	NR	MMP-14
Zhang[20]	OS	Reported	OS 1.357(1.171–1.571)	OS 1.565(1.178–1.581)	MMP-13
Kulic[21]	OS	Reported	OS 2.75(0.83–9.12)	NR	MMP-1
Song[22]	DFS	Reported	DFS 1.34(1.02–1.75)	NR	MMP-2
Talvensaari-Mattila[25]	OS,RFS	SC	OS 0.8 (0.39–1.67) RFS 1.11(0.58–2.13)	NR	MP-2
Leppa[26]	OS,DFS	Reported	OS 3.25(1.11–9.54) DFS 0.039(1.04–4.69)	NR	MMP-2
Li[27]	OS,RFS	Reported	OS MMP–2 3.350 (0.723–15.514) MMP–9 1.965 (0.519–7.436) MMP-2/MMP-9 3.144 (0.834–11.857) RFS MMP–2 3.293 (1.247–8.698) MMP–9 3.359 (1.268–8.896) MMP-2/MMP-9 2.847 (1.246–6.505)	NR	MMP-2 MMP-9
Pellikainen[28]	RFS	ReportedSC	RFS 1.81(1.09–3.0)	RFS 1.7(1.07–2.70)	MMP-9
Rahko[29]	OS,RFS	Reported SC	OS 1.08(0.50–2.00) RFS 0.8(0.5–1.4)	NR	MMP-9
Bostrom[30]	DFS	Reported	DFS 1.99(1.12–3.53)	DFS 1.81(1.01–3.22)	MMP-1
Sullu[31]	OS	Reported	OS 2.92(1.22–7.01)	NR	MMP-9
Wadowska-Jaszczyńska[32]	OS	SC	OS 4.31(0.06–304.23)	NR	MMP-2
Fernandez-Guinea[33]	OS	Reported	OS 2(1.10–3.60)	NR	MMP-9
Merdad[34]	OS	Reported	OS 1.21(0.265–6.385)	NR	MMP-9
Min[35]	OS	Reported	NR	MMP-2 2.361(1.042–5.35) MMP-9 1.418(0.658–3.056)	MMP-2 MMP-9
Chenard[36]	OS,DFS	Reported	OS 3.03 DFS 2.29	NR	MMP-11
Talvensaari-Mattila[41]	OS,RFS	SC	OS 0.68(0.08–6.14) RFS 0.76(0.11–5.73)	NR	MMP-2
Talvensaari-Mattila[42]	RFS	SC	RFS 0.62(0.18–2.18)	NR	MMP-2

Reported: reported in the article; SC: K-M survival curve; NR: not reported.

### Analysis for OS of all relevant studies

**Fig 2:**
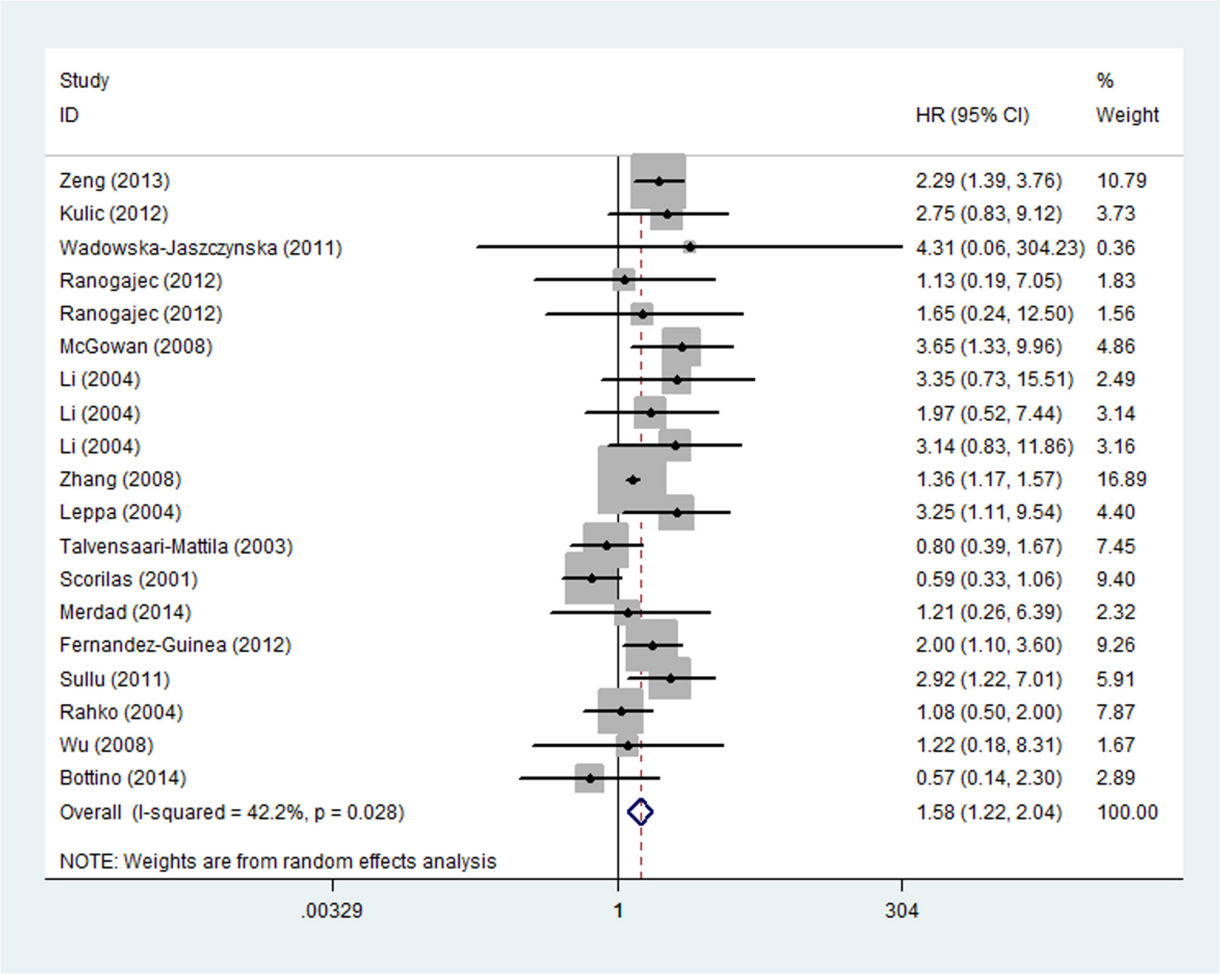
Forest Plot Showing the Association between Positive MMPs Expression and OS of Breast Cancer by univariate analysis.

**Fig 3:**
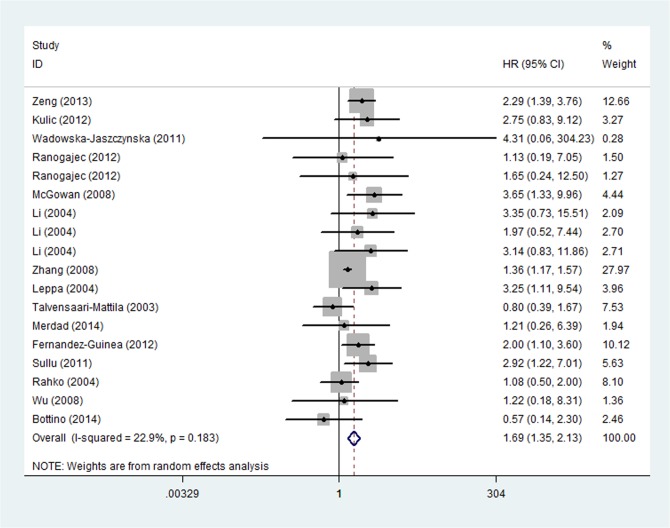
Forest Plot Showing the Association between Positive MMPs Expression and OS of Breast Cancer by univariate analysis after sensitivity analysis.

**Fig 4:**
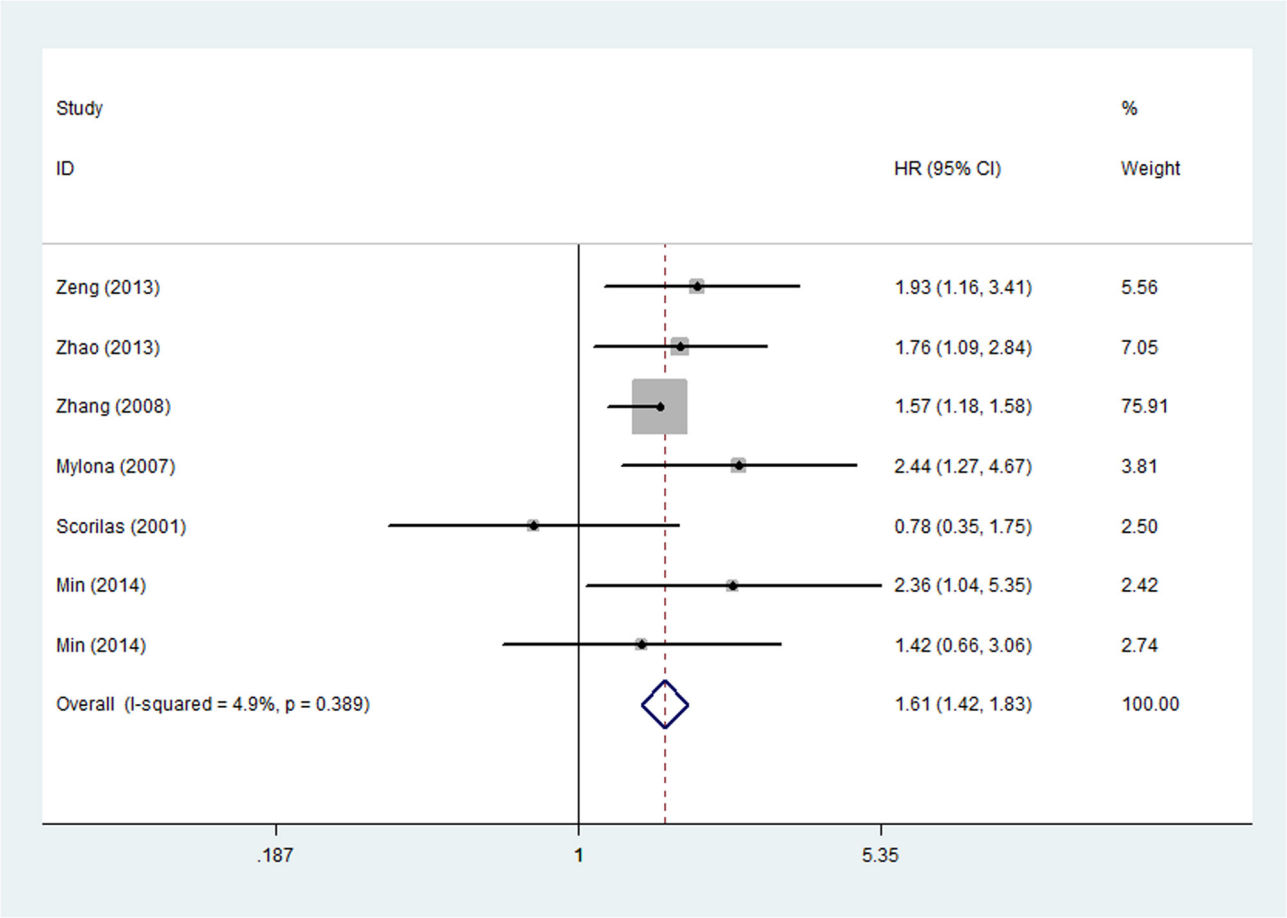
Forest Plot Showing the Association between Positive MMPs Expression and OS of Breast Cancer by multivariate analysis.

Univariate survival analysis data for OS were available in 16 studies, which included 9 studies with MMP-9 [8–10,14,27,29,31,33,34] and 5 studies with MMP-2 [11,12,15,25,27,31,32]. For the overall population, worse OS was observed among patients with MMPs overexpression (HR = 1.577, 95%CI: 1.216–2.045, P = 0.001) (Fig 2) in the 16 studies. There existed heterogeneity in our current study (P _heterogeneity_ = 0.028; I^2^ = 42.2%), so it was possible to continue categorizing the trials. The subgroup analysis was subsequently performed according to years, countries, methods, sources of MMPs, types of MMPs, HR statistics. As a result, heterogeneity was observed in subgroup analysis of types of MMPs, HR statistics, and sources of MMPs, in which heterogeneity was provided by MMP-9 (P _heterogeneity_ = 0.028, I^2^ = 51.7%), reported HR (P _heterogeneity_ = 0.01, I^2^ = 52.0%), tumor source (P _heterogeneity_ = 0.024, I^2^ = 45.7%) and tumor diameter cutoff value (2-5cm: P _heterogeneity_ = 0.020, I^2^ = 62.5%). A sensitivity analysis showed that the study by Scorila et al. was the source of the heterogeneity and removal of this study changed HR to the one in favor of MMPs-positive related to poor prognosis of breast cancer (HR = 1.694, 95%CI: 1.347–2.129, P < 0.001; P _heterogeneity_ = 0.183, I^2^ = 22.9%,) (Fig 3). Furthermore, multivariate analysis for OS including 5 [8,11,14,15,35] studies identified that breast cancer patients with MMPS overexpression had a statistically significant HR (1.611, 95%CI: 1.419–1.830, P < 0.001) and no significant heterogeneity (P _heterogeneity_ = 0.389, I^2^ = 4.9%) (Fig 4).

### Analysis for OS of MMP-9 expression

**Fig 5:**
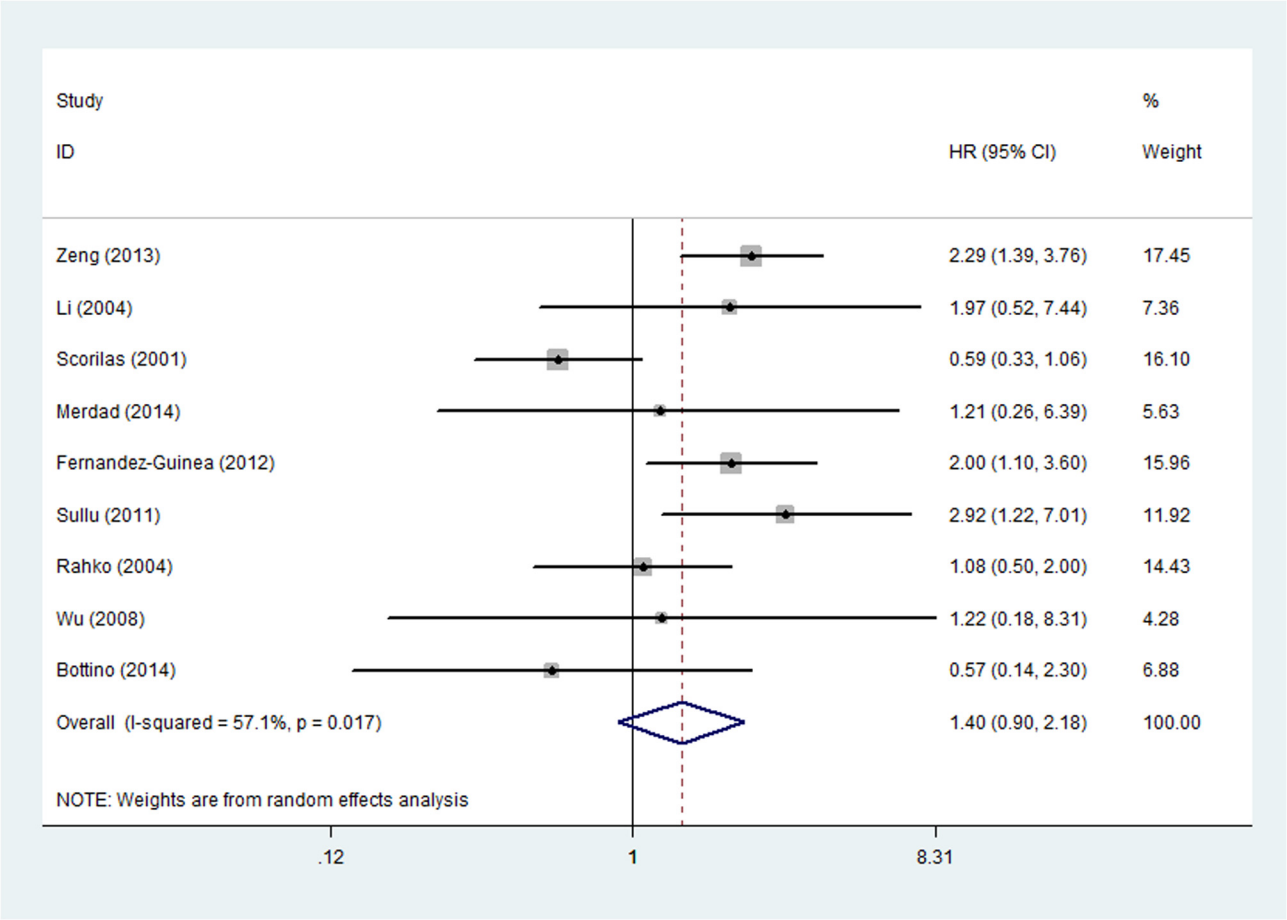
Forest Plot was Designed to Visualize the Association between Positive MMP-9 Expression and OS of Breast Cancer by univariate analysis.

**Fig 6:**
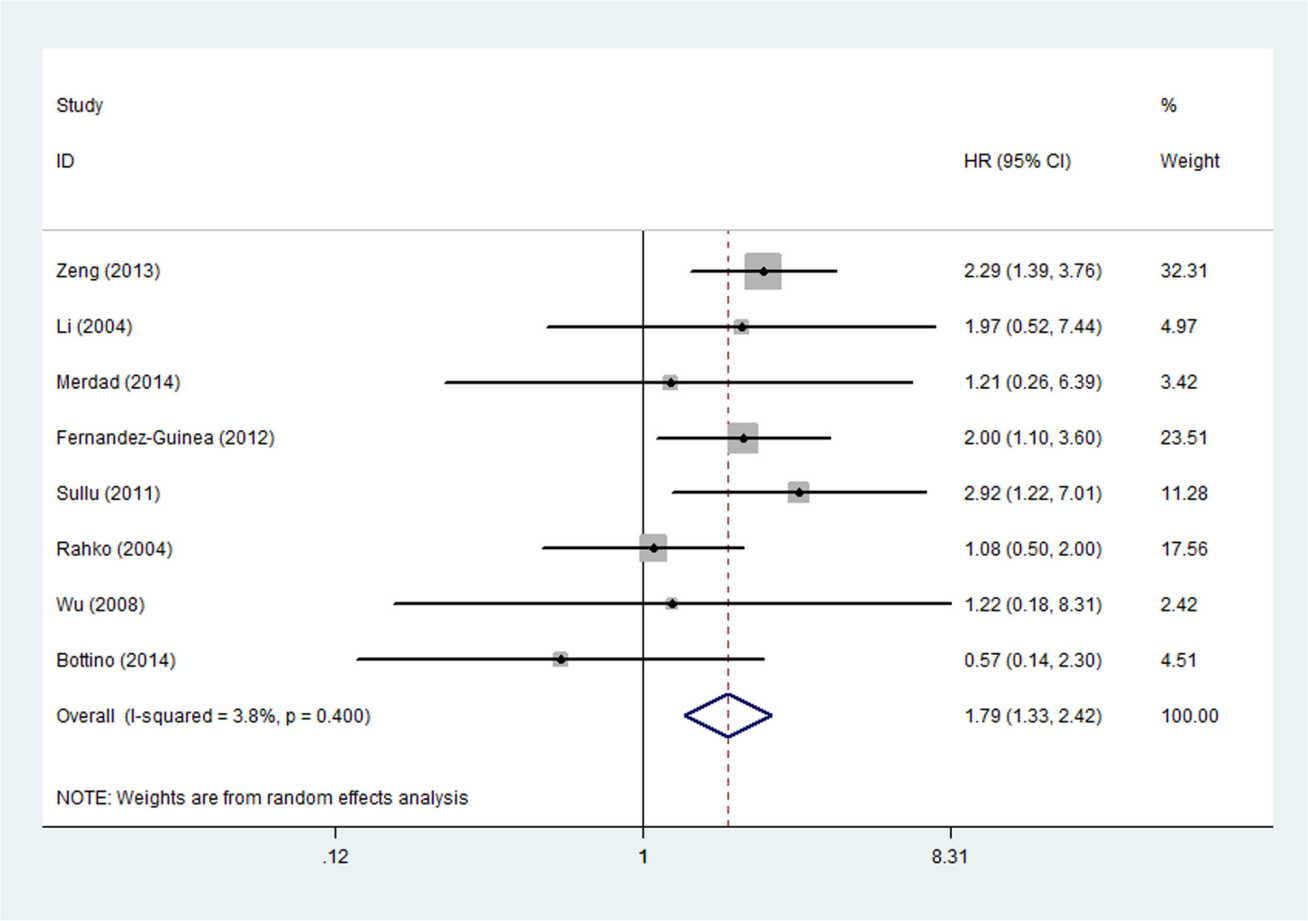
Forest Plot was Designed to Visualize the Association between Positive MMP-9 Expression and OS of Breast Cancer by univariate analysis after sensitivity analysis.

The relationship between OS and MMP-9 up-regulation was analyzed in nine studies by univariate analysis [8–10,14,27,29,31,33,34]. The pooled HR was 1.404 (95%CI: 0.903–2.181, P = 0.131) and heterogeneity existed (P _heterogeneity_ = 0.017, I^2^ = 57.1%) (Fig 5). The subgroup analysis was performed according to the publish year, countries, methods and tumor diameter cutoff value, respectively. According to the result of subgroup analysis, the source of heterogeneity may come from the subgroup of IHC (P _heterogeneity_ = 0.017, I^2^ = 61.1%) and the subgroup with HR reported within the study (P _heterogeneity_ = 0.018, I^2^ = 58.5%). At a further step analysis of sensitivity, HR was statistically changed to 1.794 (95%CI: 1.330–2.420, P < 0.001) and the heterogeneity disappeared (P _heterogeneity_ = 0.400, I^2^ = 3.8%) when study by Scorlia et al. was excluded (Fig 6). Multivariate analysis was performed in 4 studies [8,11,14,15,35] and the outcome demonstrated that the pooled HR was 1.678 (95%CI: 1.216–2.315, P = 0.002) with no significant heterogeneity (P _heterogeneity_ = 0.253, I2 = 25.2%).

### Univariate analysis for RFS

**Fig 7:**
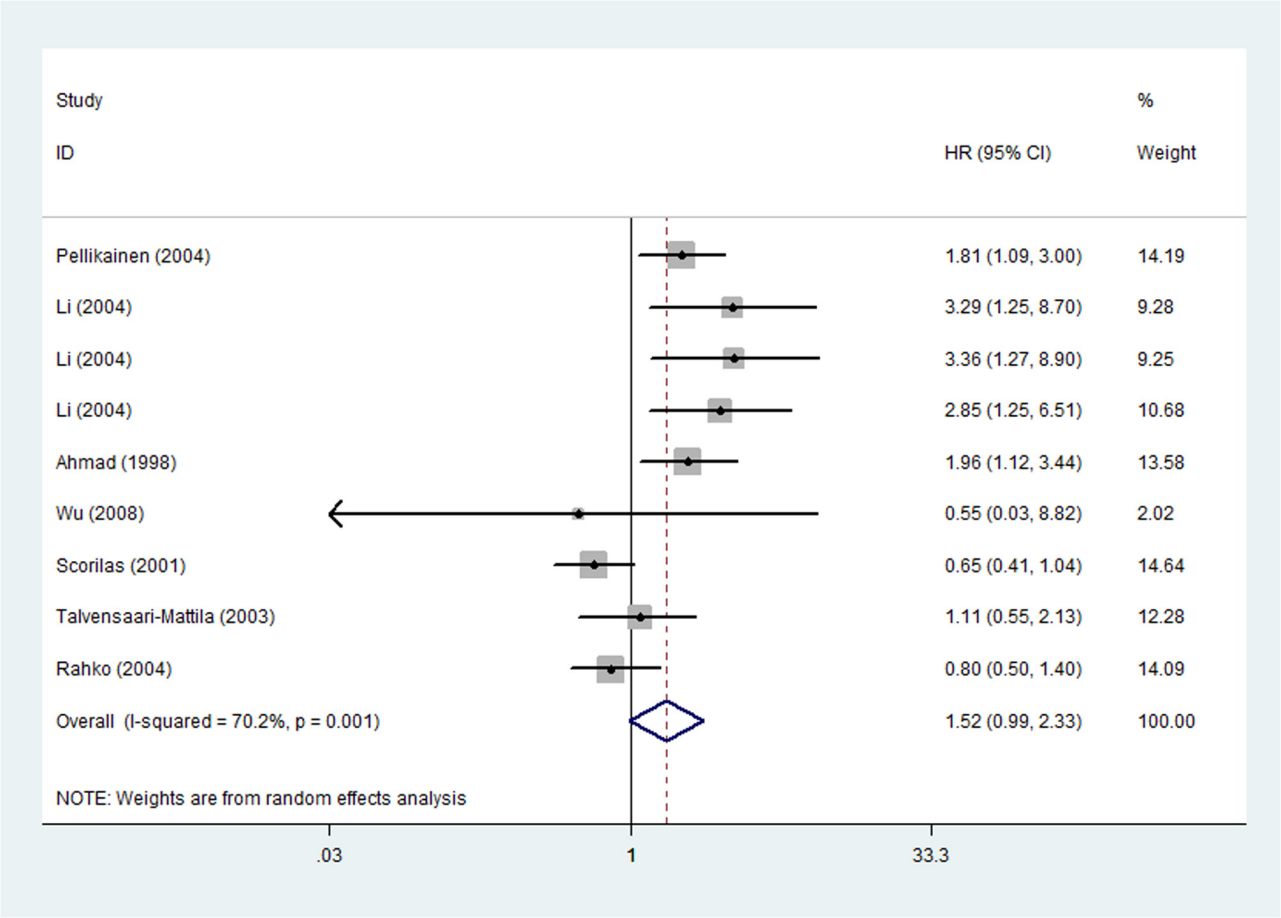
Forest Plot Showing the Association between Positive MMPs Expression and RFS of Breast Cancer by univariate analysis.

The pooled HR of 7 studies [8,9,17,25,27,29,32] relevant to RFS was 1.516 (95%CI: 0.987–2.328, P = 0.057) and there existed heterogeneity (P _heterogeneity_ = 0.001,I^2^ = 70.2%) (Fig 7). The heterogeneity became absent and the pooled HR changed to be statistically significant after removal of two studies (Scorilas et al.[8] and Rahko et al.[29]), which were calculated by sensitivity analysis.

### Univariate analysis for other factors associated with the survival

The results showed that no relationship was observed between MMP-2 positive patients and OS (HR = 1.400, 95%CI: 0.610–3.209, P = 0.427) [12,25–27,32]. However, overexpression of MMPs in serum was significantly in relation to worse OS in breast cancer patients (HR = 1.630, 95%CI: 1.065–2.494, P = 0.025) [9,13,21,22,26]. What’s more, the pooled HR (95%CI) of DFS was statistically significant (HR = 1.718, 95%CI: 1.301–2.268, P < 0.001). Additionally, there was no significant heterogeneity in the three groups involved in this paragraph.

### Publication bias

**Fig 8:**
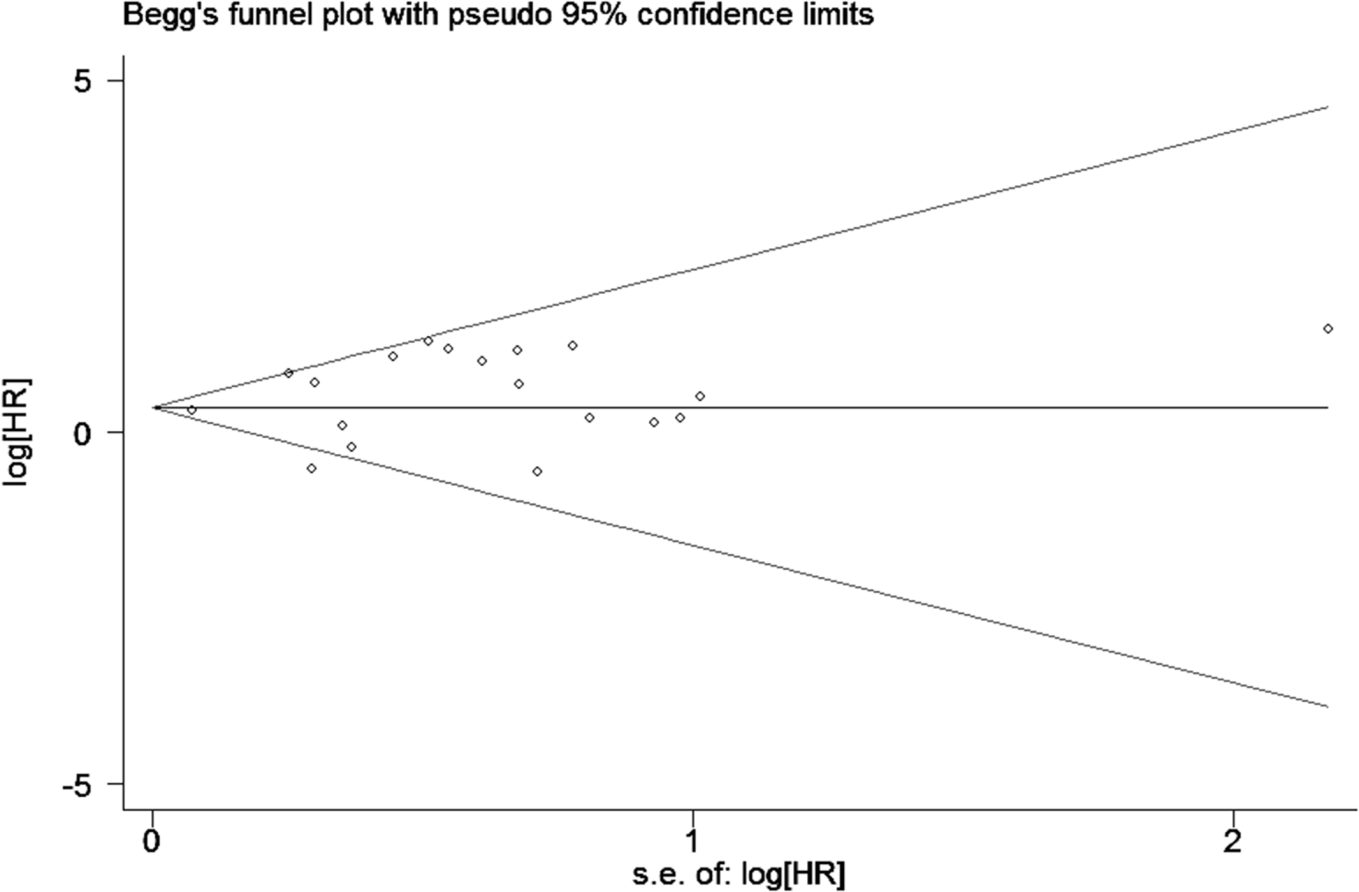
Begg’s funnel plot for publication bias test on studies assessing MMPs expression and OS of breast cancer by univariate analysis.

Publication bias was assessed by Begg’s funnel plot and Egger’s test. The combined results of Begg’s funnel plots (Fig 8) and P values of the Egger’s test (P = 0.281) suggested no obvious publication bias in univariate analysis of OS of MMPs-overexpressed patients.

### Systematic review

Some articles referred to the prognosis of patients with MMPs positive expression were not given the sufficient information, so they were described as follows. Positive staining (>30%) of MMP-11 expression was obviously associated with shorter OS and DFS in breast cancer, and the influence was even more significant in patients with node-positive metastasis [36]. Another report demonstrated that high expression level of MMP-11 existed in invasive breast tumors, especially in invasive ductal carcinomas, which might provide some clues for prognosis [37]. Savinov et al. [38] concluded that expression of MMP-26 was inversely related with breast tumor stage in ductal carcinoma and in favor of better survival.

## Discussion

### Summary of the results

Previously, two meta-analyses published by Song et al. [16] and Chen et al. [39] have verified that MMP-9 and MMP-2 overexpression predicted higher risk for OS and RFS in patients with breast carcinoma. Compared with the preceding meta-analysis, our meta-analysis is the first one to demonstrate that MMPs family may be biomarkers for worse prognosis on breast cancer patients, including not only the aforementioned MMP-2 and MMP-9, but also MMP-1, MMP-11, MMP-13 and MMP-14. Furthermore, 27 studies have been involved in the current meta-analysis, and the number of patients was 4944, which was more than 15 studies involved with 2344 patients in the report of Song et al. [16] and 9 studies composed of 1614 patients that in the study reported by Chen et al. [39]. More importantly, our current study has evaluated the association between MMPs expression and the survival time of breast cancer patients by using univariate and multivariate analysis. With this method, the current study gains advantages over that studied by Song et al [16] and Chen et al. [39] which were only analyzed by multivariate analysis or the combination of univariate and multivariate analysis. Therefore, our current study could provide more powerful evidence for the prognostic value of MMPs in breast cancer. In the present study, the pooled HR (95%CI) of univariate analysis for OS changed from 1.577 (1.216–2.045) to 1.694 (1.347–2.129) in patients with overexpression of MMPs. And the heterogeneity disappeared after excluding the study of Scorilas et al.[8], which might contribute to the heterogeneity. By sensitivity analysis, heterogeneity was possibly due to the alteration in the baseline features of patients (cutoff of tumor diameter, type of MMPs expression and the reported way to calculate HR and 95%CI). However, because the available information was limited, we could not clearly clarify why the study reported by Scorilas et al.[8] caused heterogeneity. At the same time, the multivariate analysis also provided evidence to identify the worse OS of MMPs expression on breast cancer patients and no heterogeneity emerged.

In the sub-group analysis, with the exclusion of the source (Scorila et al.[8]) of heterogeneity, the pooled HR and 95% for MMP-9-positive patients significantly changed from 1.404 (0.903–2.181) to 1.794 (1.330–2.420) and no heterogeneity was observed. While pooled HR (95%CI) valued by multivariate analysis further verified the worse prognosis of MMP-9 positive in breast cancer patients. This outcome was consistent with a previous meta-analysis (Song[16] et al.). Except for the association between MMP-9 up-regulation and the prognosis of breast cancer patients, Merdad et al. [34] also detected that mRNA of MMP-9 overexpression was particularly associated with the Invasive ductal Carcinoma (IDC) of breast cancer.

However, it is controversial whether MMP-2 positivity was relevant with worse OS in breast cancer patients. In our study, worse OS was not observed in patients with MMP-2 positivity by univariate analysis. While, there were other studies demonstrated that MMP-2 positivity was relevant to a poor prognosis in breast cancer [18], especially in postmenopausal patients with node-positive breast carcinoma [34]. In comparison with MMPs-negative patients in serum, the opposite ones had shorter time for survival (HR = 1.630, 95%CI: 1.065–2.494). And Talvensaari-Mattila et al.[40–42] found for the first time that hematogenous metastasis was in correlation with MMP-2 positivity (P = 0.03), which may further reveal the poor prognosis suggested by MMP-2 expression in serum. Meanwhile, the univariate analysis results also demonstrated that MMPs expression could worsen DFS and RFS in breast cancer patients. However, as the articles involved in the analysis of DFS, RFS and OS (MMPs-positive in serum) were less than 5, the results remained controversial. It was needed to investigate the association between overexpression of MMPs and DFS, RFS and OS (MMPs-positive in serum) in MMPs positive breast cancer patients at a further step.

In addition, some researches also partially showed the mechanism involved in the prognosis of MMPs positive breast cancer patients. There is one study suggesting that an MMP-26–mediated intracellular pathway that targets estrogen receptorβ (ERβ) perhaps contributes favorably to the survival of ERα/β-positive patients [34]. Pierre et al. [36] concluded that MMP-11 might participate in breast cancer progression by providing cancer cells with stromal environment required for expansion outside the epithelial compartment. Another article also identified that high level of MMP-11 was in relation to invasive breast carcinoma and worsened prognosis [37]. The study reported by Folgueira et al. [43] have demonstrated that positive expression of MMP-13 in cancer-associated fibroblasts (CAFs) is in relation to regional metastasis not lymph node involvement, which is in controversial with the research studied by Zhang et al.[20] included in our meta-analysis. The co-expression of MMP-2 and MMP-9 was identified by Li et al. [27]and Ranogajec et al. [12] to be in association with patients’ RFS (HR = 2.847, 95%CI: 1.246–6.505, P = 0.013; HR = 1.65, 95%CI: 0.235–12.5, P = 0.004), but not overall survival (P = 0.091). Another study reported by Min et al. have demonstrated that although the co-expression of tumoural MMP-2 and -9 was not associated with OS(P = 0.204), it was significantly related to poor DFS (p = 0.003)[35]. Min et al. [35]have also testify that stromal MMP-2 and tumoural MMP-9 co-expression was in relation to poor OS via univariate and multivariate analysis(HR: 3.199, 95%CI:1.334–7.671, P_univariate_: 0.001, P_multivariate_:0.009). In conclusion, MMPs might predict worse prognosis by promoting tumor metastasis, invasion and growth in breast cancer through degrading cytokines and cell adhesion molecules and stimulating angiogenesis and growth factors.

### Limitation

By using an extensive search strategy, we attempted to ensure the completeness of the included results. However, as we excluded non–English studies, some relevant studies may be missed from this meta-analysis. Besides, some HRs and 95%CIs were calculated by data reported in text or survival curve. Thus, there might be some individual factors in the way of calculating survival curve, which might affect the results. To ensure the homogeneity of studies included, we mainly focused on the studies which proposed the clinical practice. Therefore, some studies might have been excluded, as a result of strict inclusion and exclusion criteria. Although there were some articles associated with DSS and PFS in addition to OS, it was not feasible to calculate the correct results. As only one study was relevant to data on DSS (HR: 0.532, 95%CI: 0.164–1.726, P = 0.021) and PFS (HR: 1.824, 95%CI: 1.22–2.965, P = 0.015) in the articles included [37,38]. The two studies [37,38] were described in systemic review other than meta-analysis. One of the main limitations of the studies was the lack of consensus about the definition of the cutoff for positivity and tumor diameter of MMPs expression. Articles involved in this meta-analysis were almost applied to demonstrate the influence of MMP-9 and MMP-2 expression, only one study on MMP-1, MMP-13 and MMP-14 and two studies for MMP-11, respectively. As a result, this meta-analysis focuses on effects of MMP-9 and MMP-2 expression on prognosis for breast cancer. There is no sufficient evidence to identify that the expression of MMPs has worse OS (HR = 1.52, 95%CI: 1.165–2.015).

## Conclusion

In conclusion, our meta-analysis demonstrates the poor prognostic significance of MMPs overexpression in breast cancer patients. Among the MMPs observed, MMP-9 seems to act as predictors for worse prognosis in breast cancer. Thus, early and exact detection of the expression level of MMP-9 may provide effective introductions for the prognostic therapy of MMP-9 positive breast cancer patients. The most interesting finding is that MMPs in serum have a negative impact on OS, which may propose a safer and more validating method to detect the expression of MMPs and then provide evidence for early diagnosis. Given small numbers of articles in association with MMPs expression in serum, more researches are required to identify the availability of this new method. Besides, many other MMPs, for example MMP-1,-13, -14, may have influence on prognosis of breast cancer patients. Therefore, further studies with larger size are needed to verify the impact of these MMPs on prognosis of breast cancer. CONSORT Checklist S1 Table.

## Supporting Information

S1 File The example of search strategy. (PPT)

S2 File Reasons for excluding the 52 full text. (DOC)

S1 Table CONSORT Checklist. (DOC)
