# Supplementary material for: Overexpression of MMP Family Members Functions as Prognostic Biomarker for Breast Cancer Patients: A Systematic Review and Meta-Analysis
Source: PLoS One. 2015 Aug 13;10(8):e0135544. doi: 10.1371/journal.pone.0135544 (PMC4535920; doi:10.1371/journal.pone.0135544)
Supplement: S1 File — (PPT) [file pone.0135544.s001.ppt]

## Slide 1
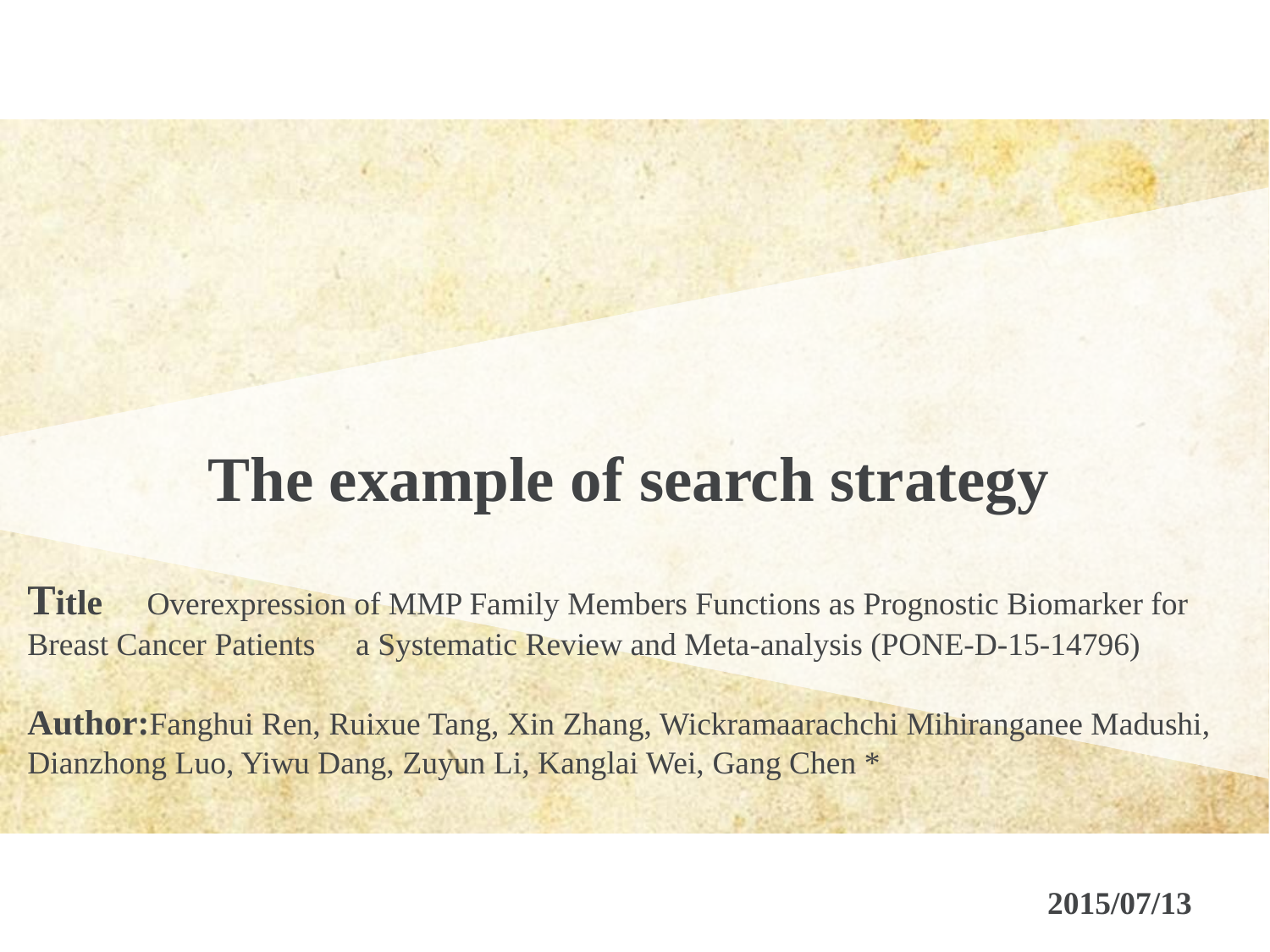

# The example of search strategy
Title：Overexpression of MMP Family Members Functions as Prognostic Biomarker for Breast Cancer Patients：a Systematic Review and Meta-analysis (PONE-D-15-14796)
Author:Fanghui Ren, Ruixue Tang, Xin Zhang, Wickramaarachchi Mihiranganee Madushi, Dianzhong Luo, Yiwu Dang, Zuyun Li, Kanglai Wei, Gang Chen *
2015/07/13

## Slide 2
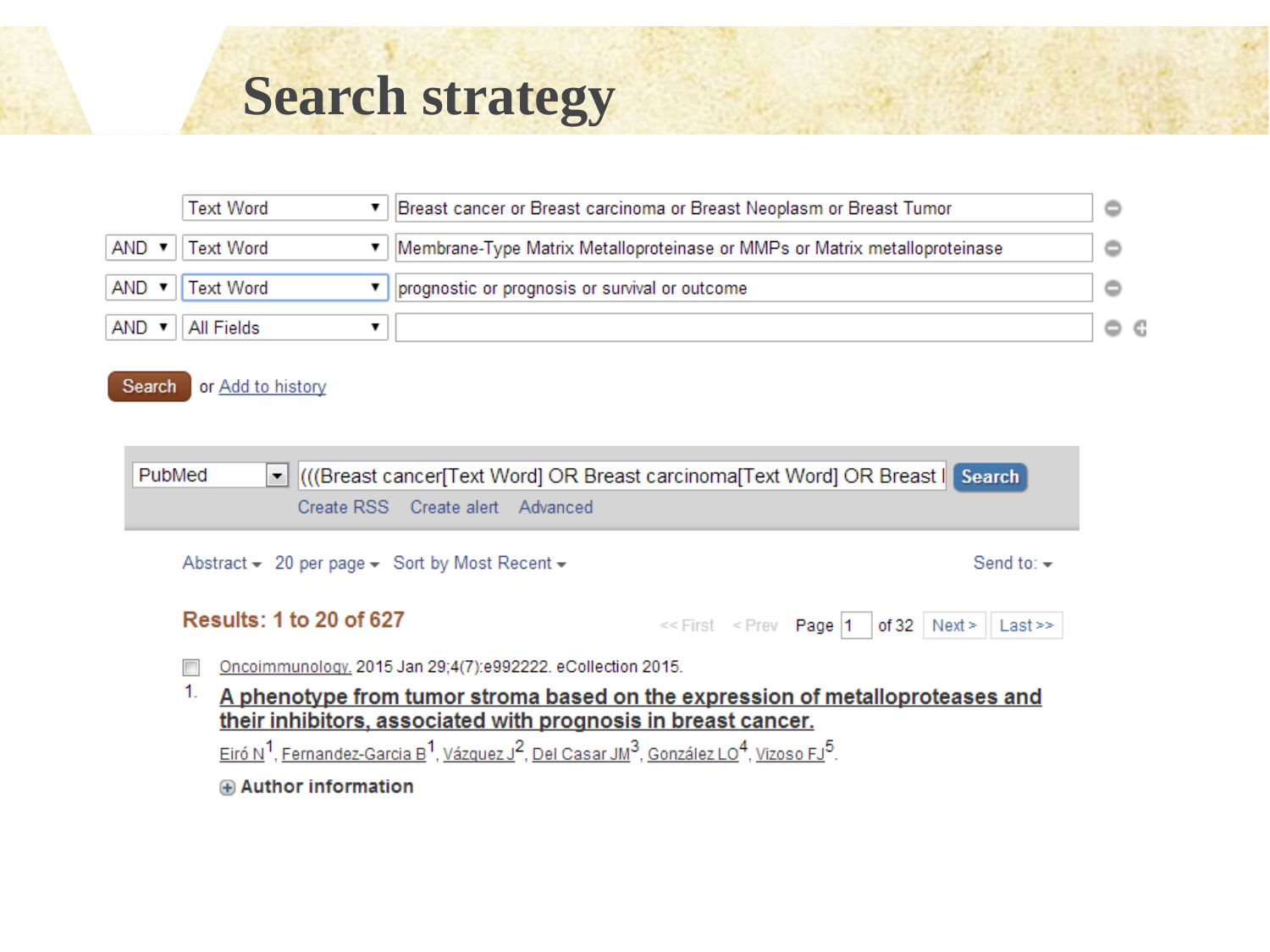

# Search strategy

## Slide 3
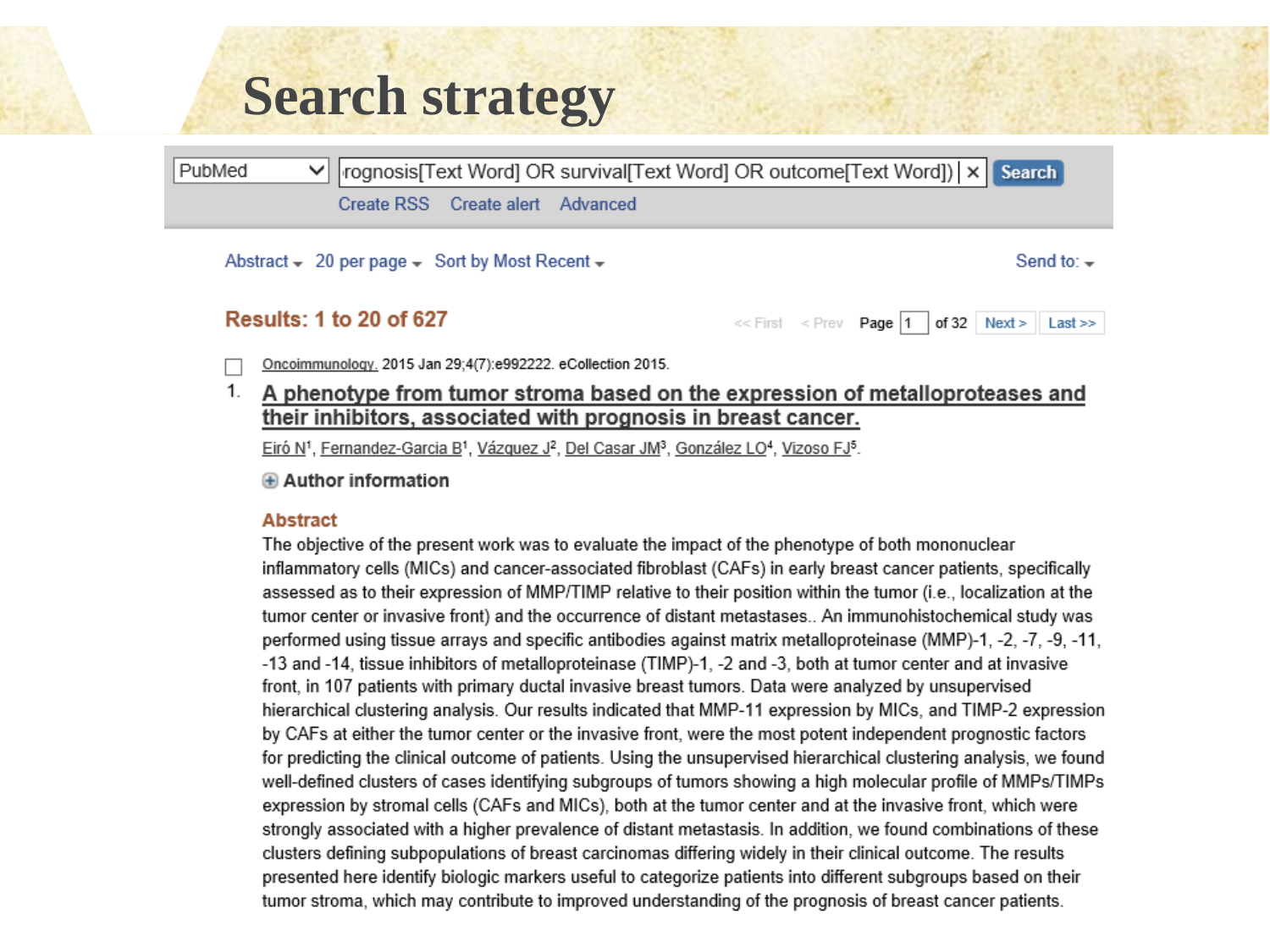

# Search strategy

## Slide 4
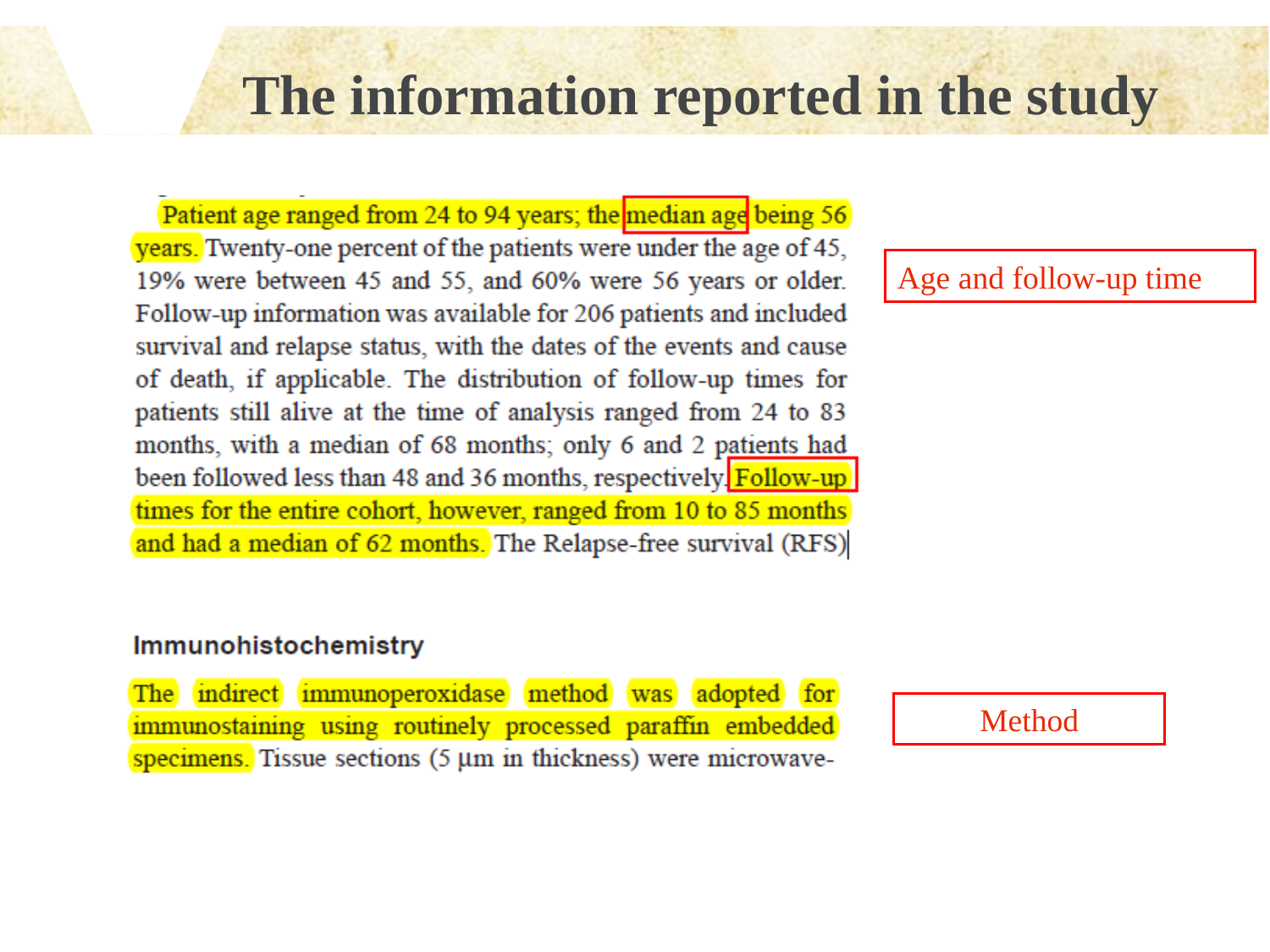

# The information reported in the study
Age and follow-up time
Method

## Slide 5
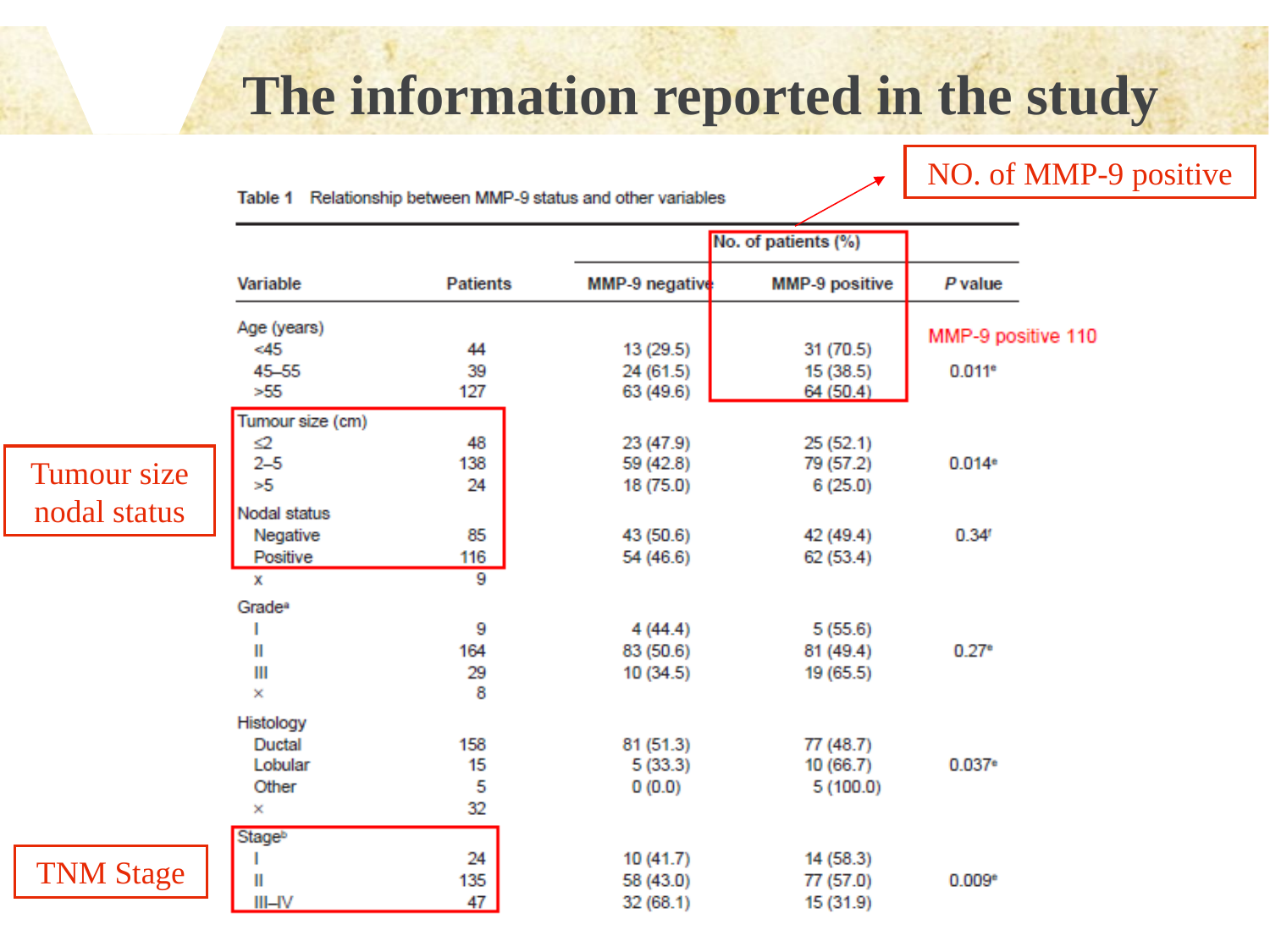

# The information reported in the study
NO. of MMP-9 positive
Tumour size
nodal status
TNM Stage

## Slide 6
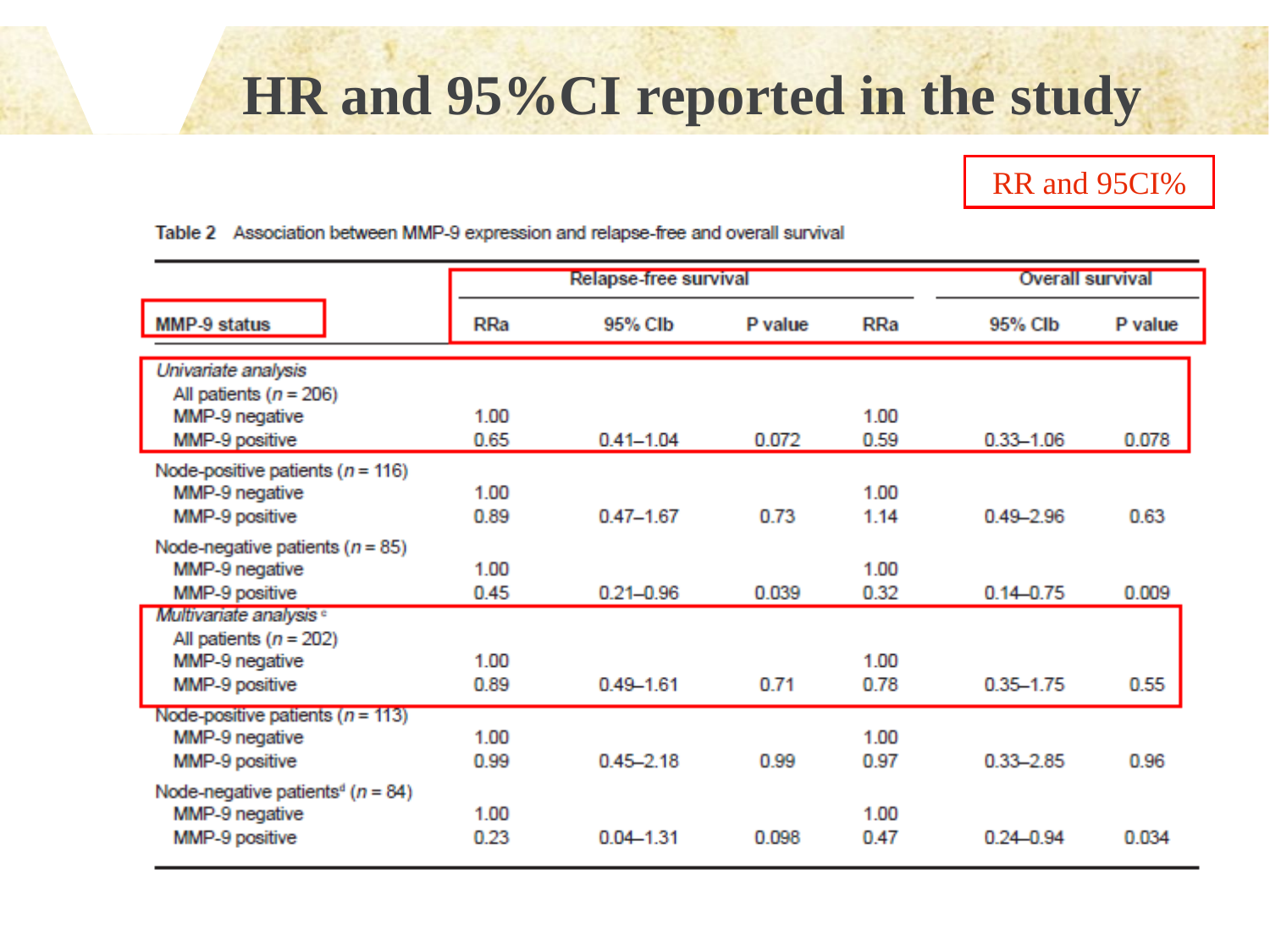

# HR and 95%CI reported in the study
RR and 95CI%

## Slide 7
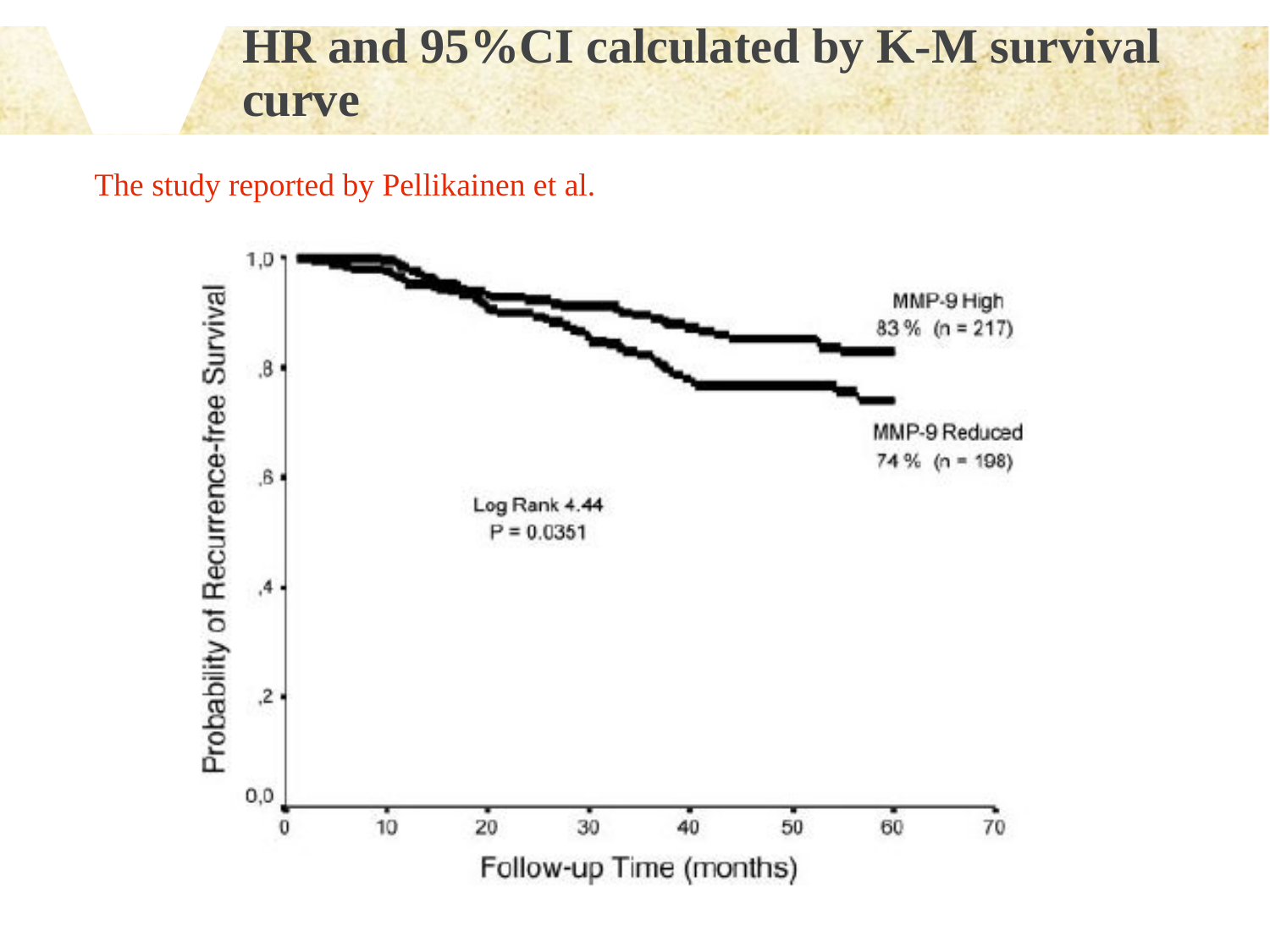

# HR and 95%CI calculated by K-M survival curve
The study reported by Pellikainen et al.

## Slide 8
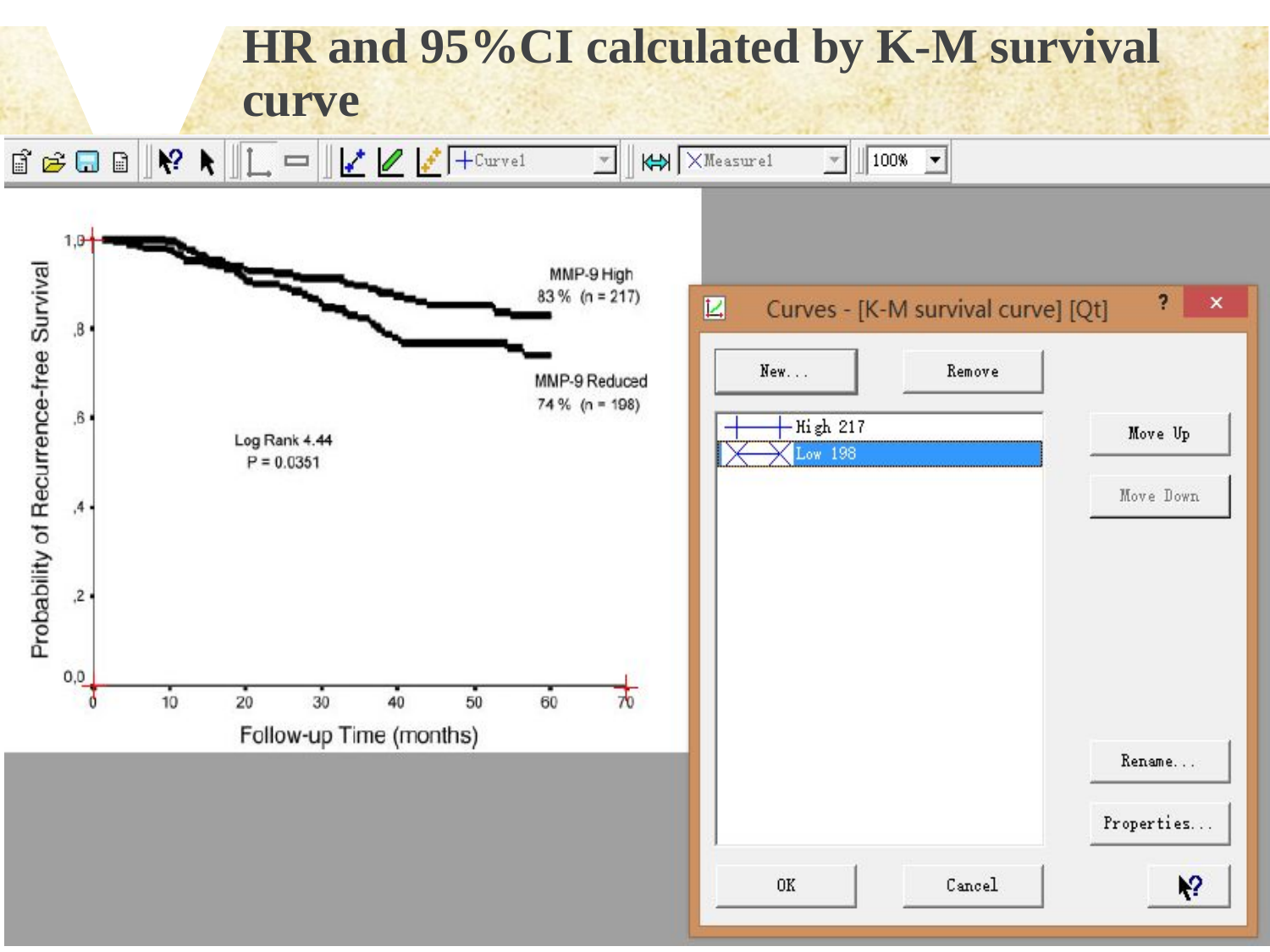

# HR and 95%CI calculated by K-M survival curve

## Slide 9
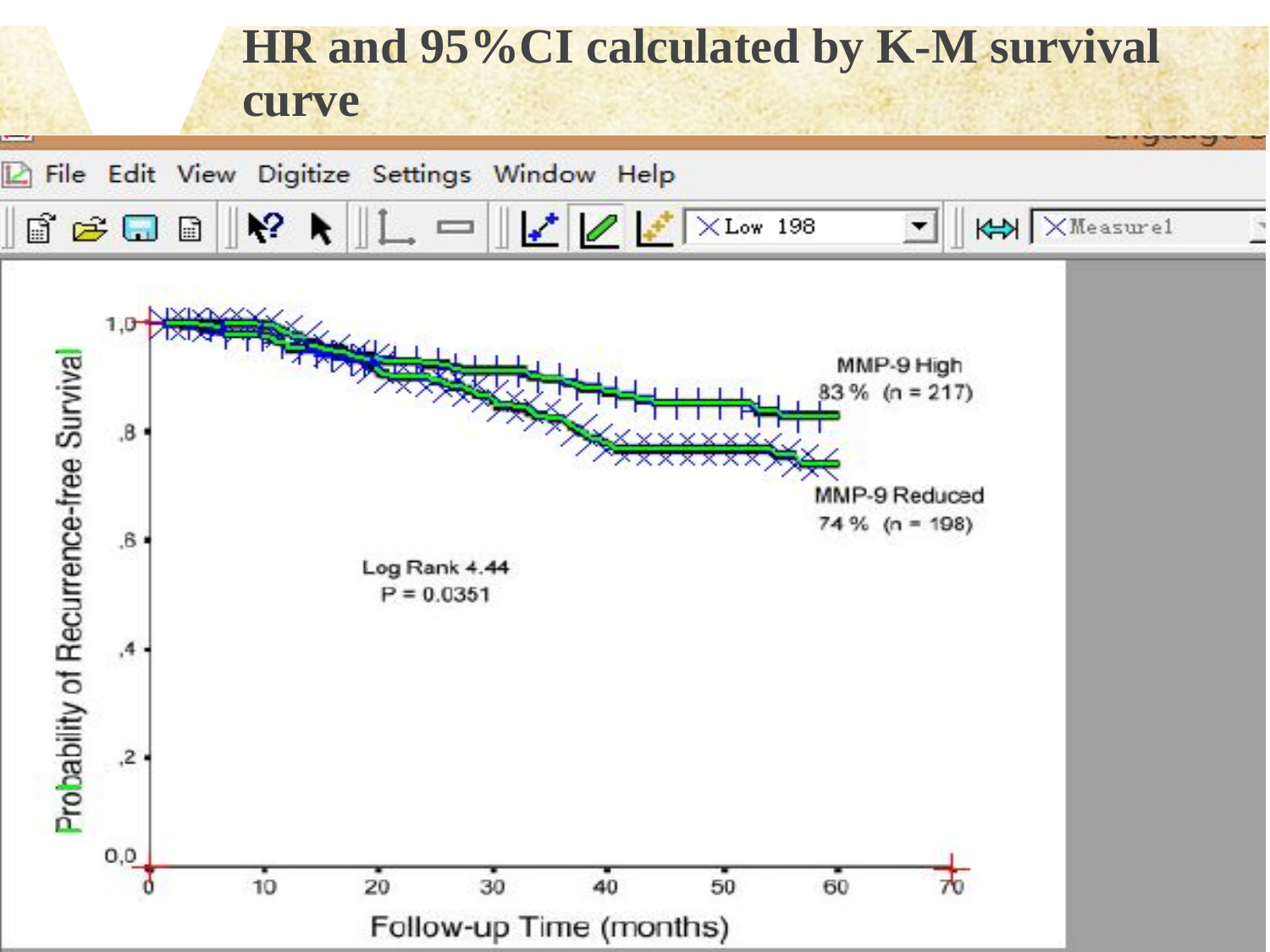

# HR and 95%CI calculated by K-M survival curve

## Slide 10
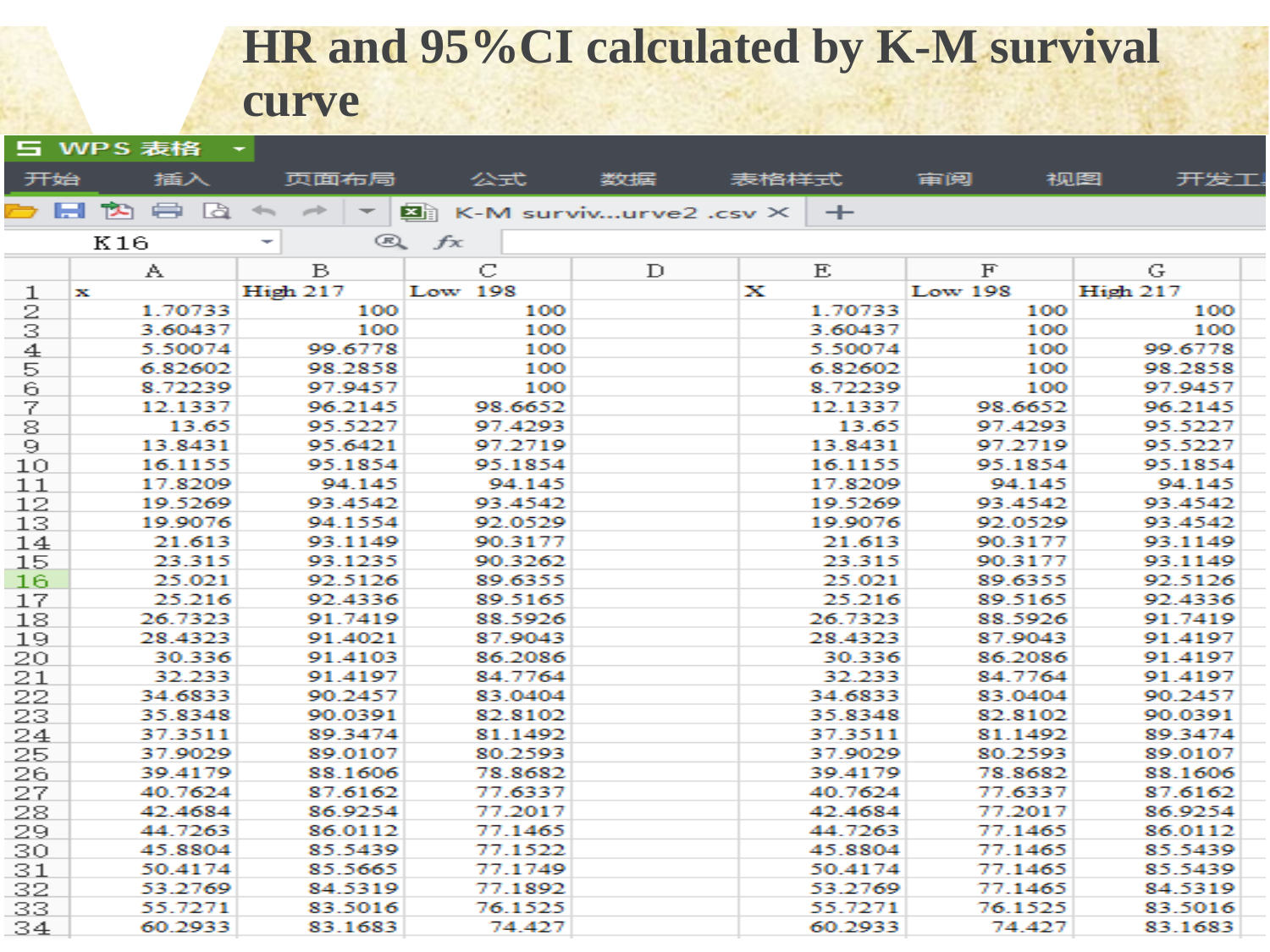

# HR and 95%CI calculated by K-M survival curve

## Slide 11
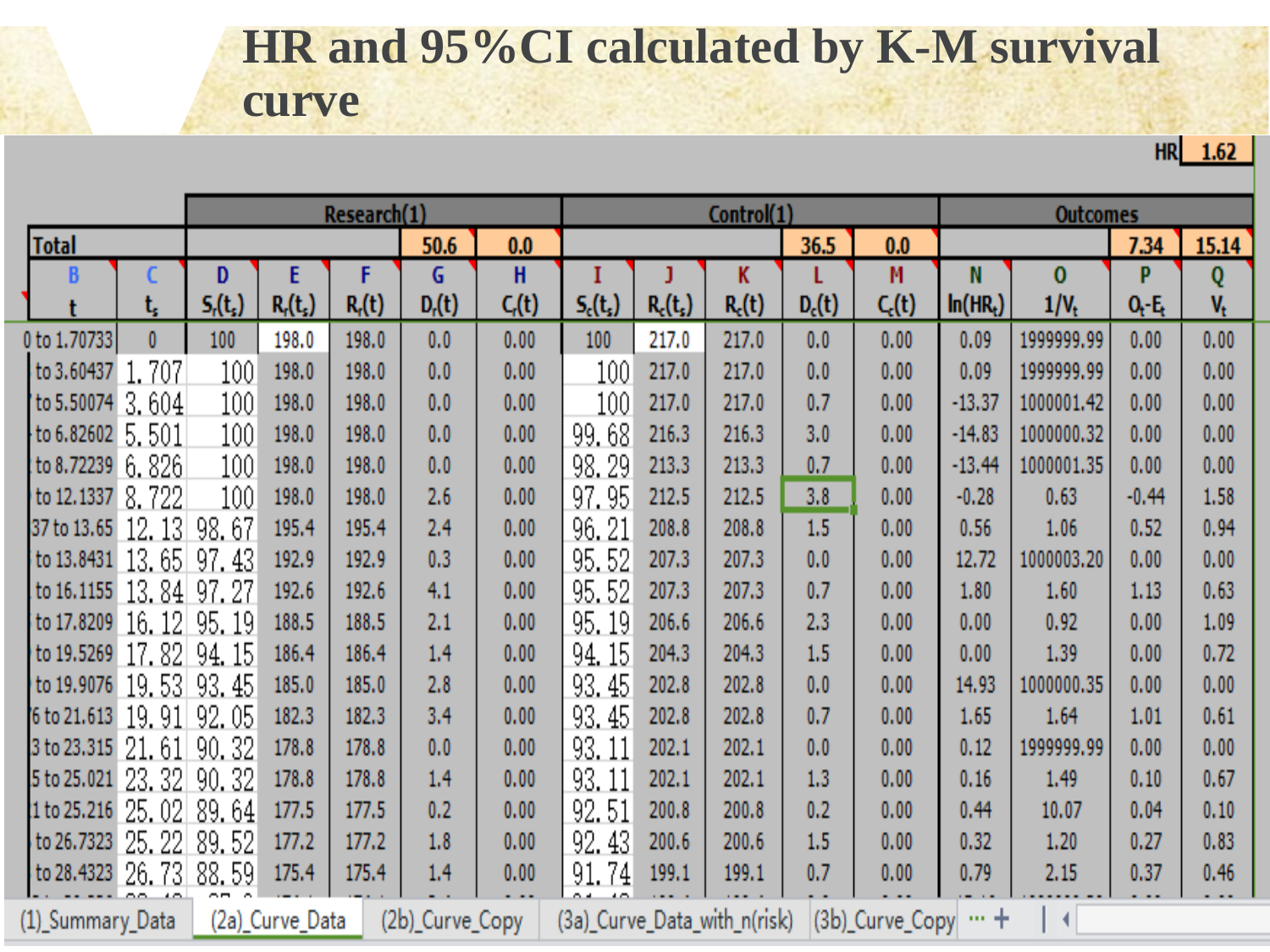

# HR and 95%CI calculated by K-M survival curve

## Slide 12
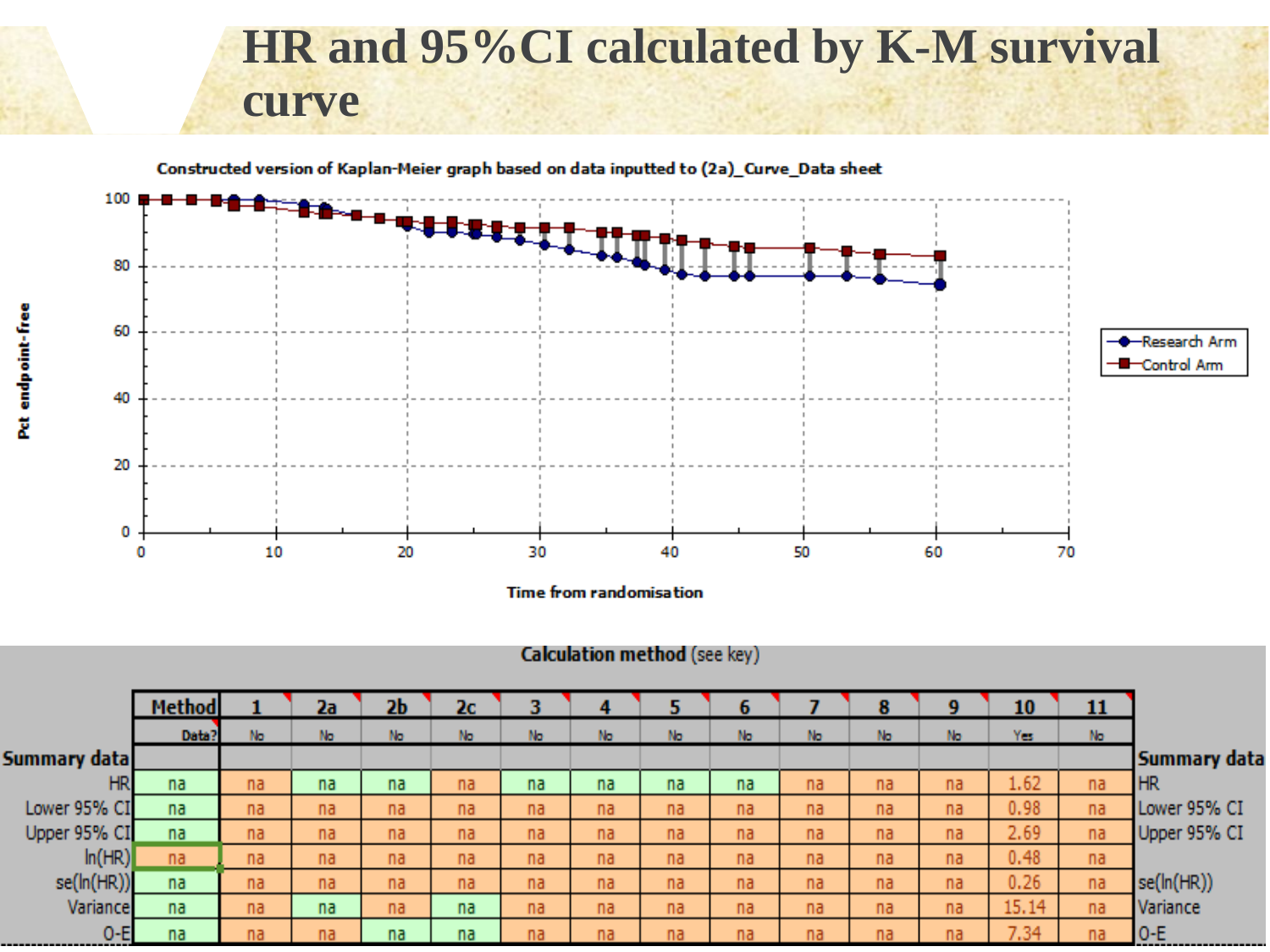

# HR and 95%CI calculated by K-M survival curve

## Slide 13
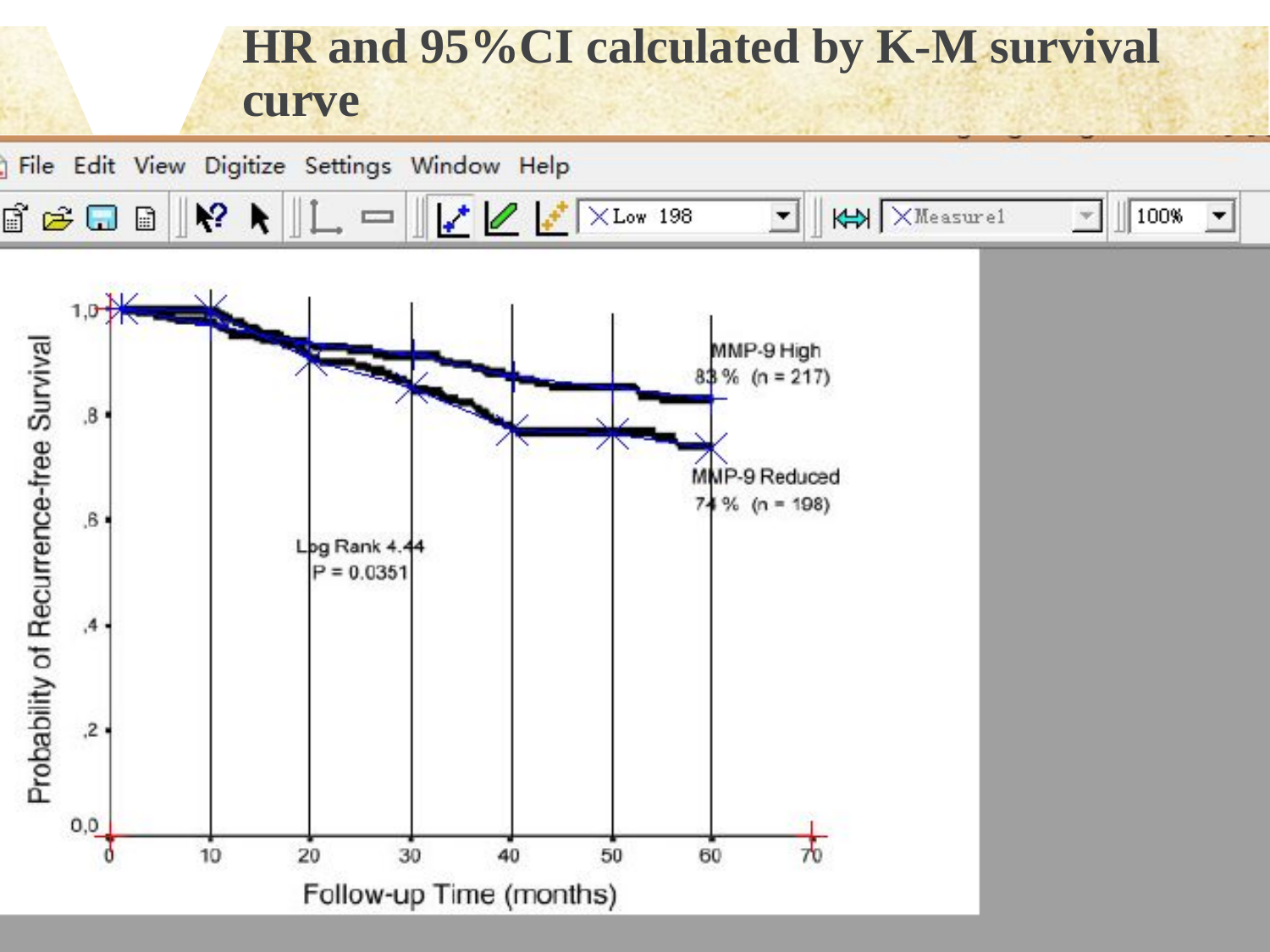

# HR and 95%CI calculated by K-M survival curve

## Slide 14
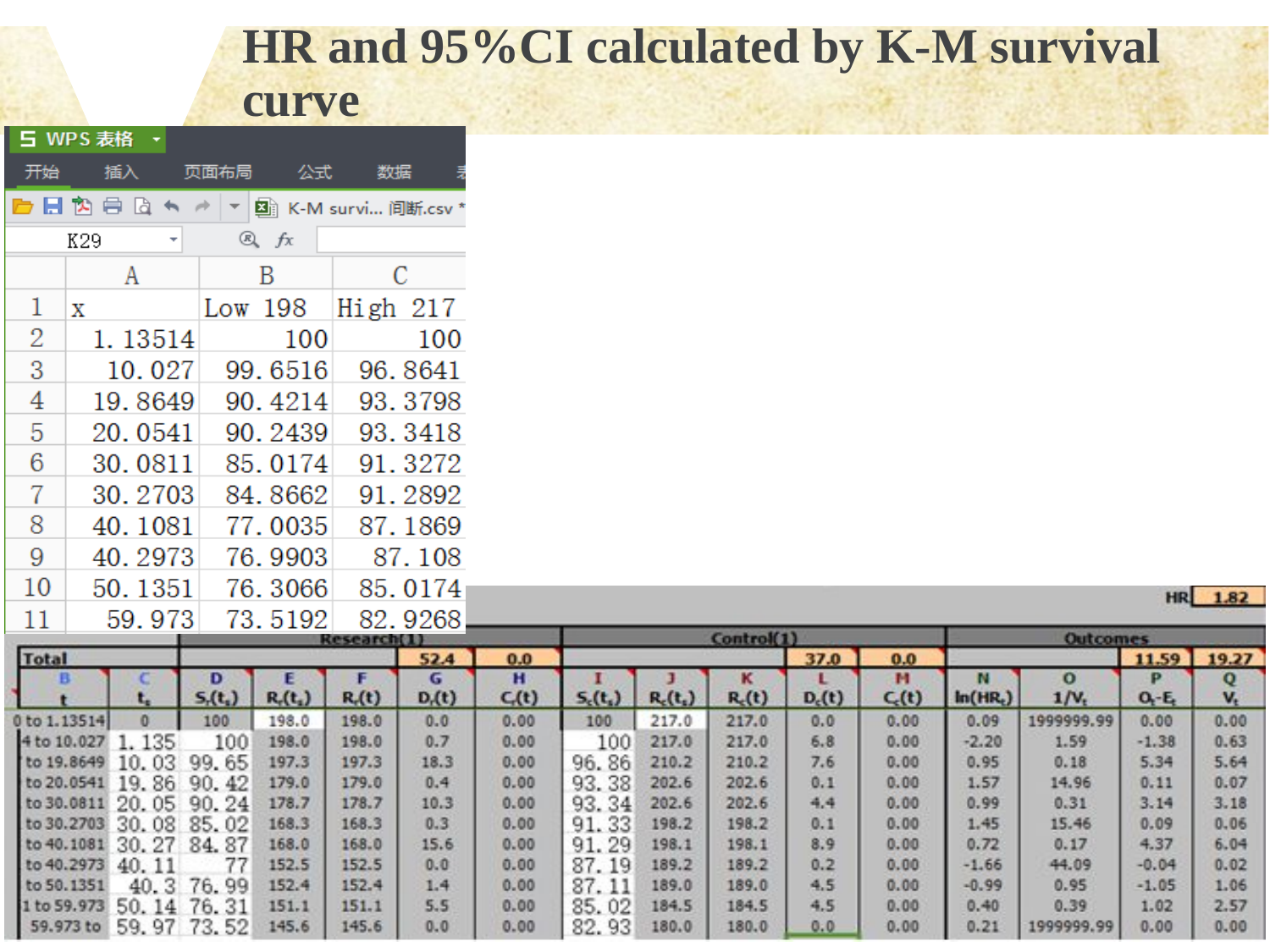

# HR and 95%CI calculated by K-M survival curve

## Slide 15
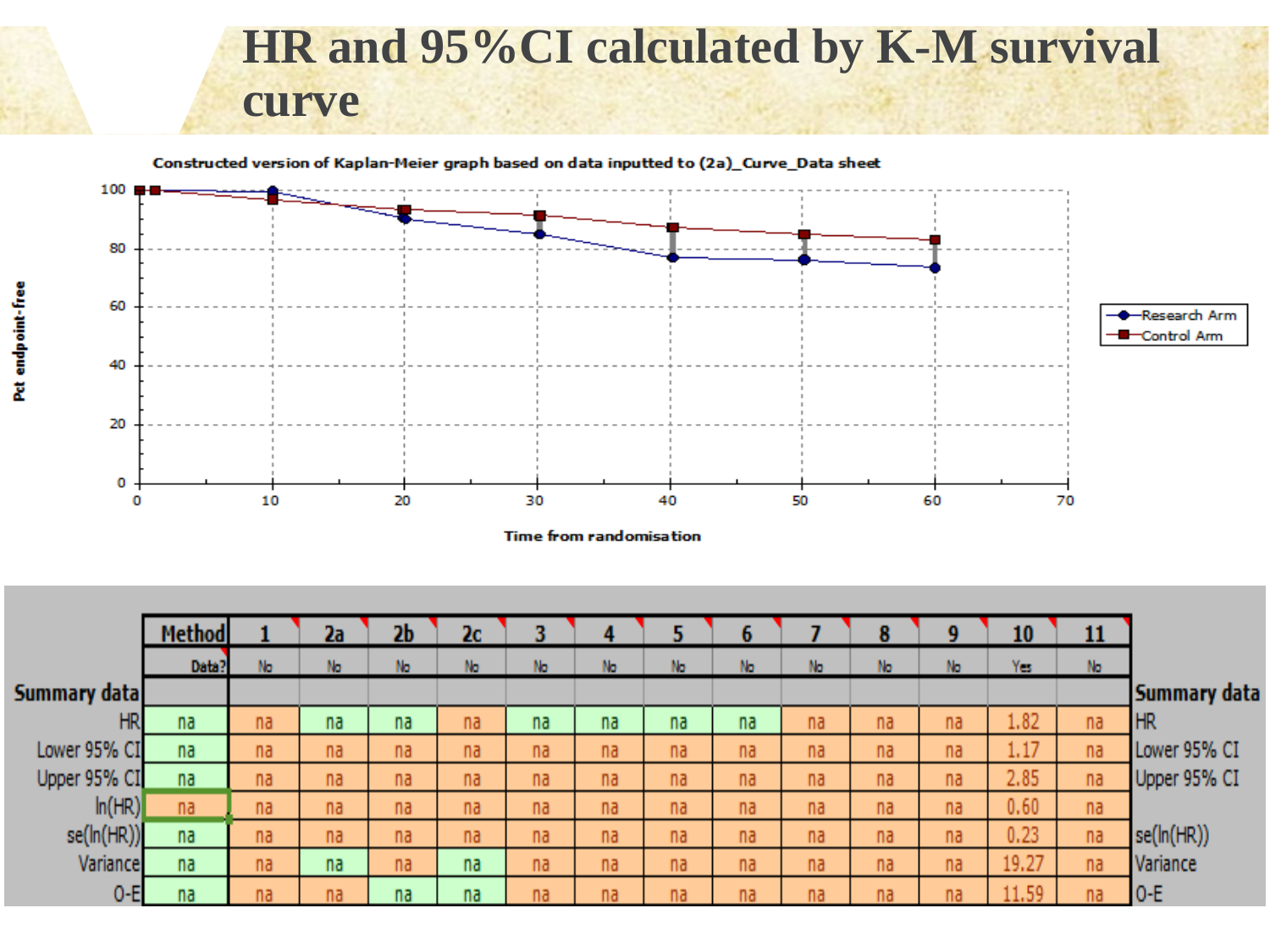

# HR and 95%CI calculated by K-M survival curve

## Slide 16
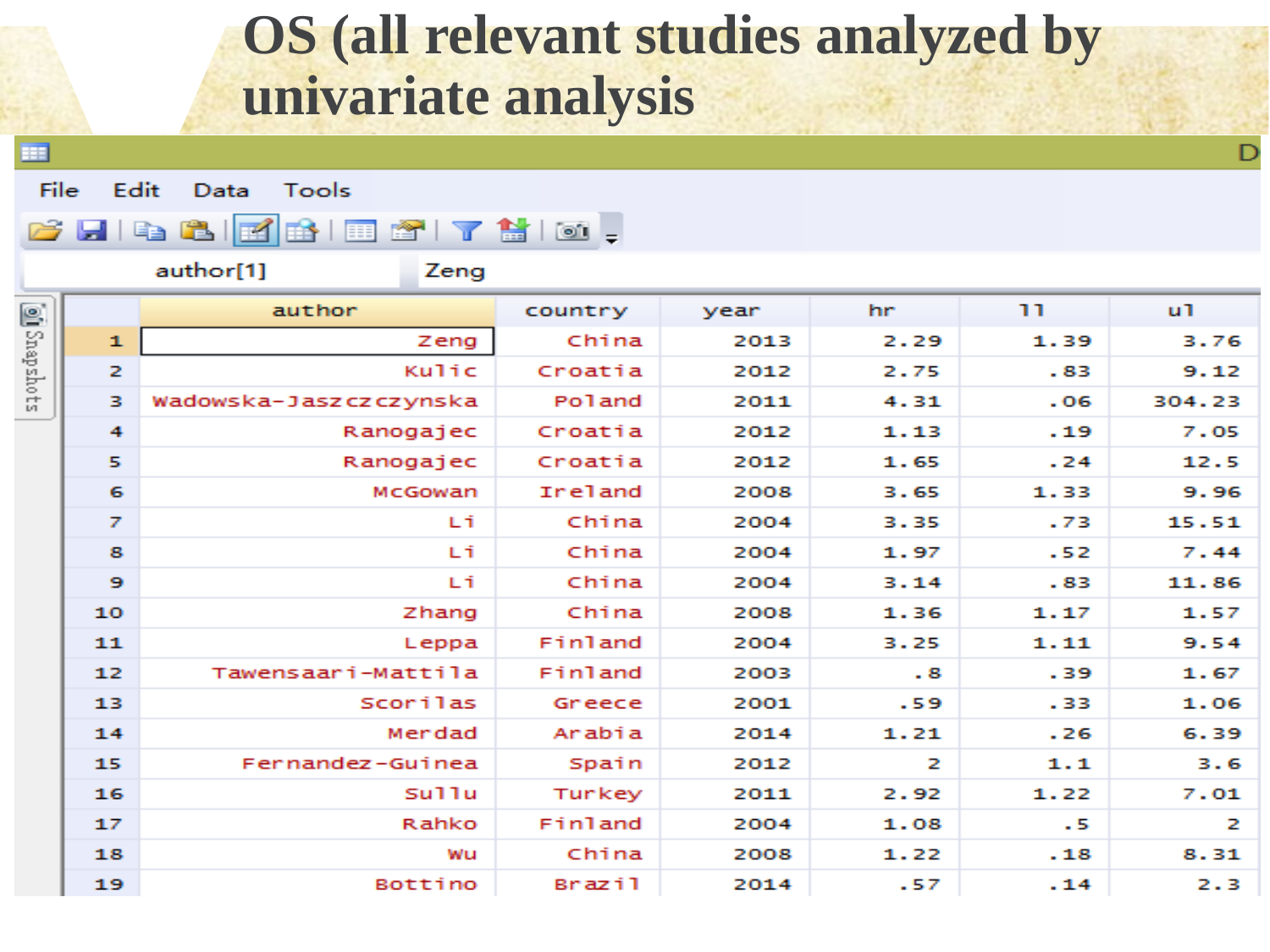

# OS (all relevant studies analyzed by univariate analysis）

## Slide 17
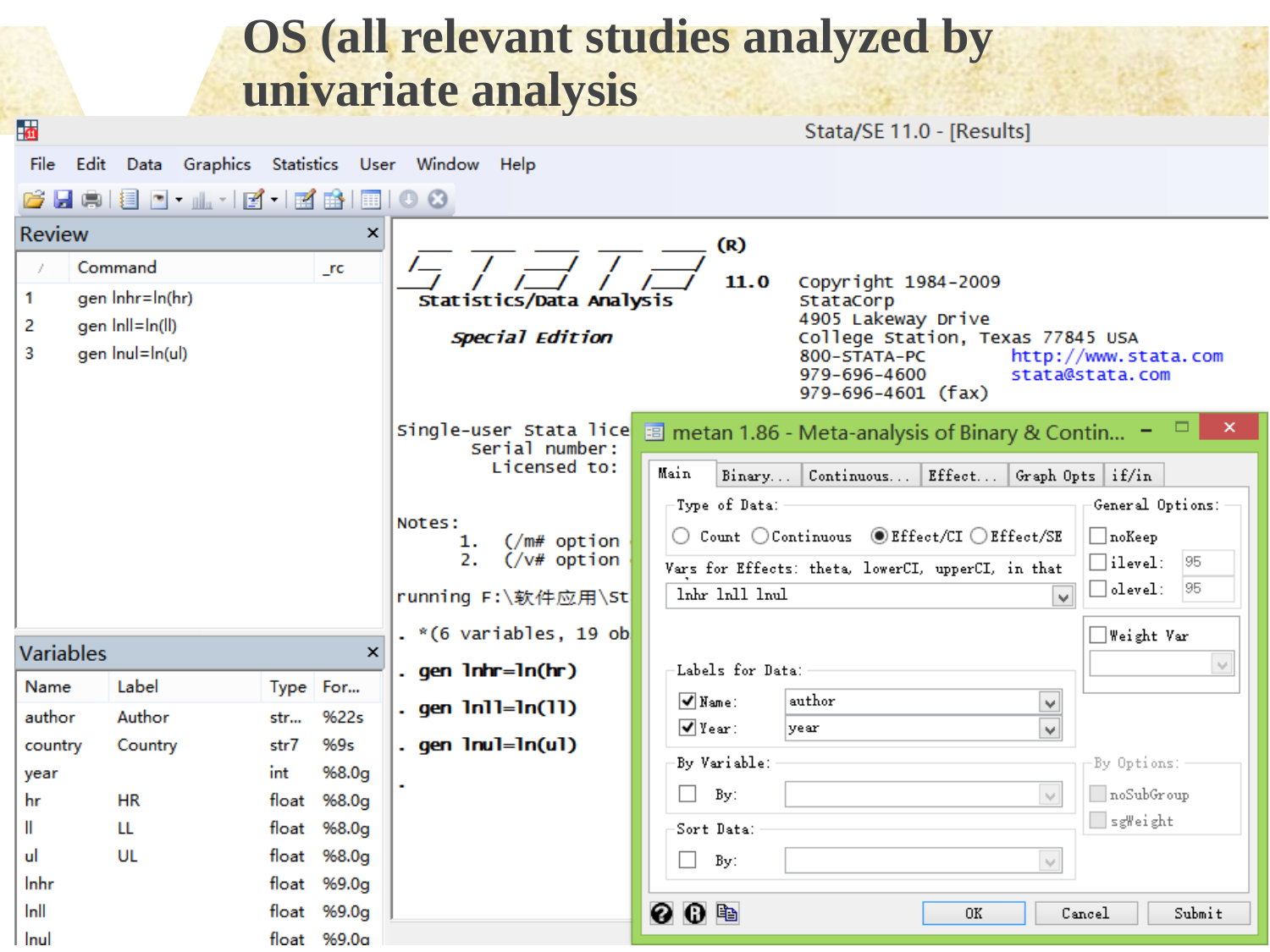

# OS (all relevant studies analyzed by univariate analysis）

## Slide 18
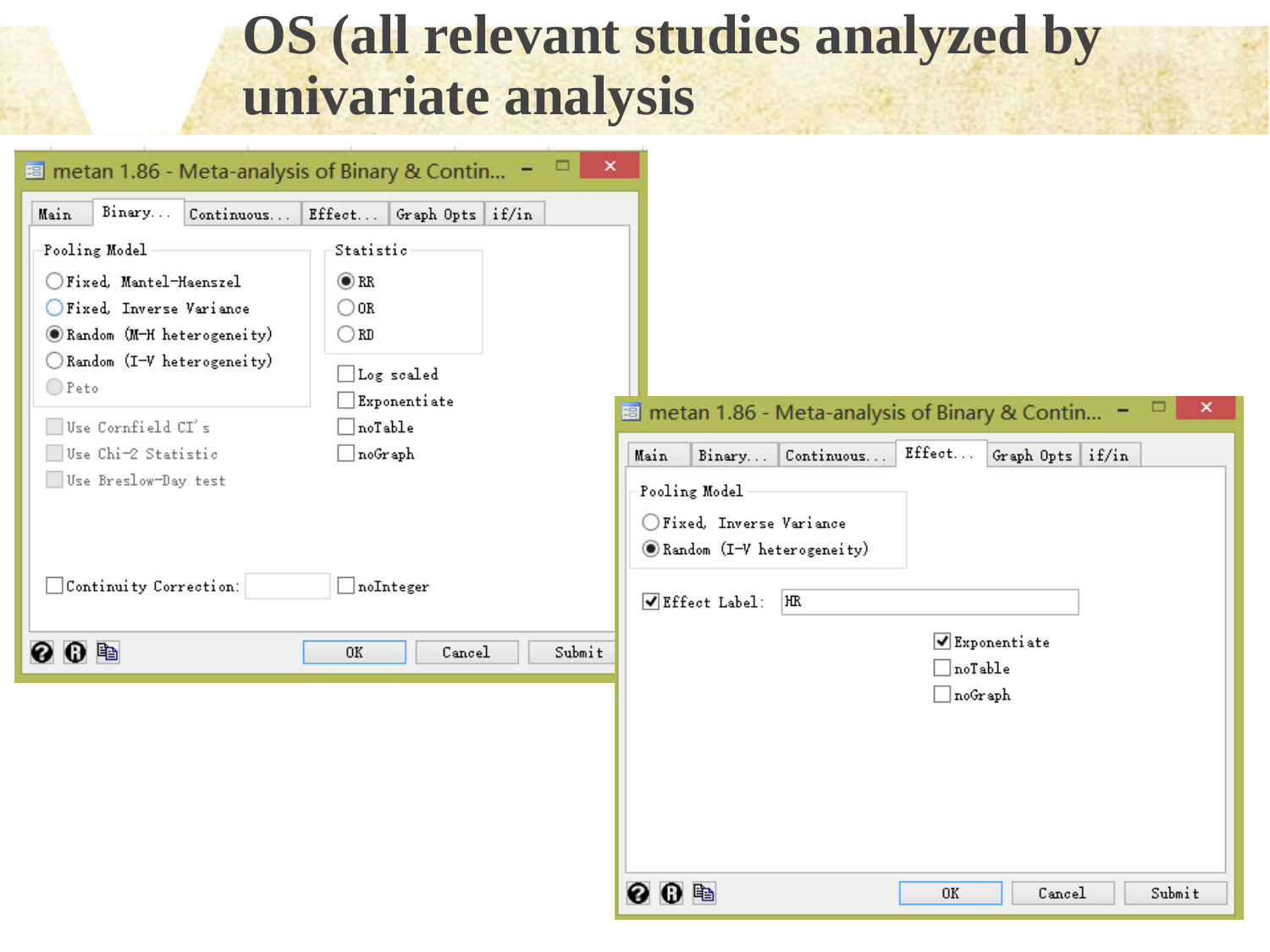

# OS (all relevant studies analyzed by univariate analysis）

## Slide 19
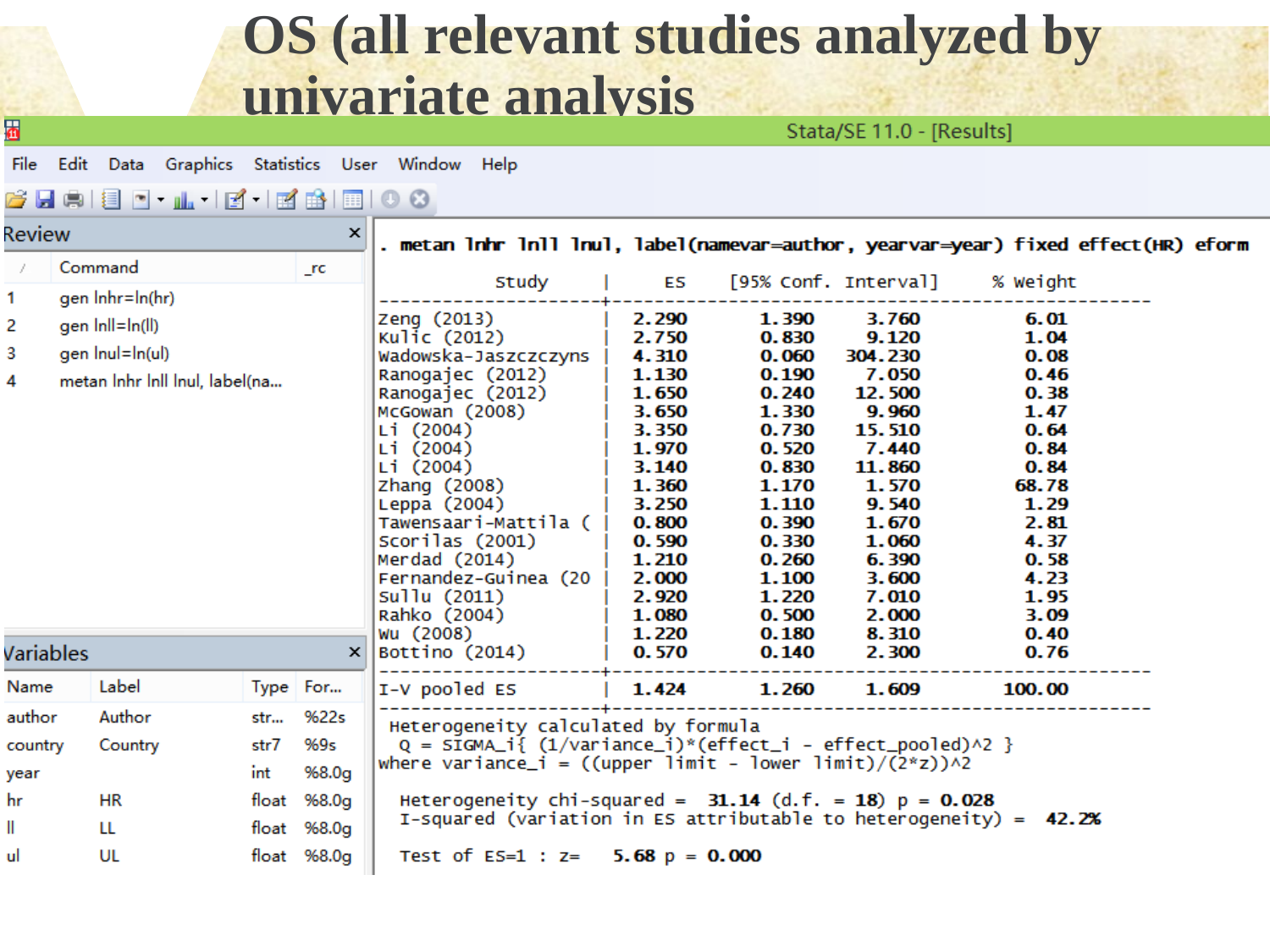

# OS (all relevant studies analyzed by univariate analysis）

## Slide 20
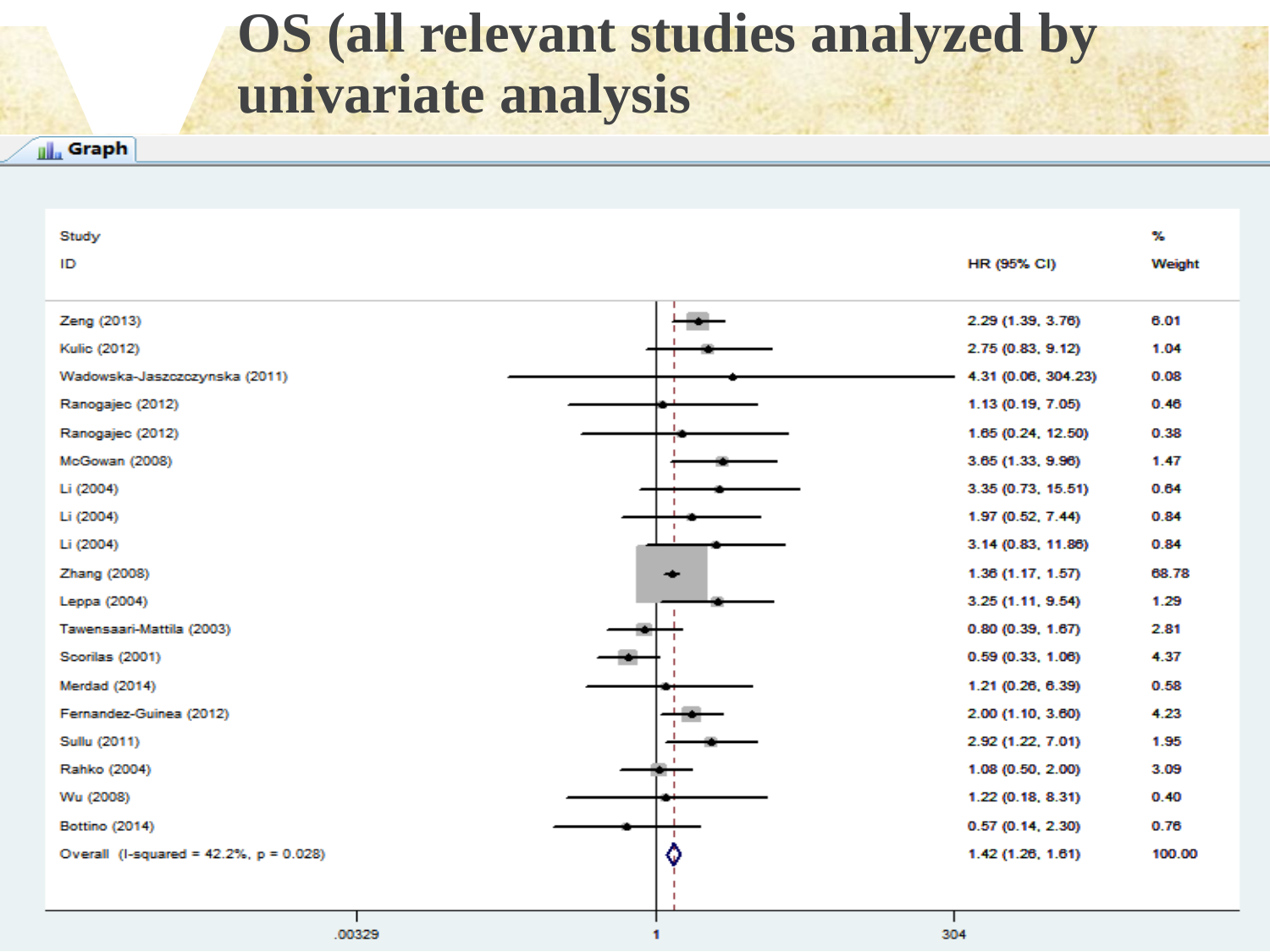

# OS (all relevant studies analyzed by univariate analysis）

## Slide 21
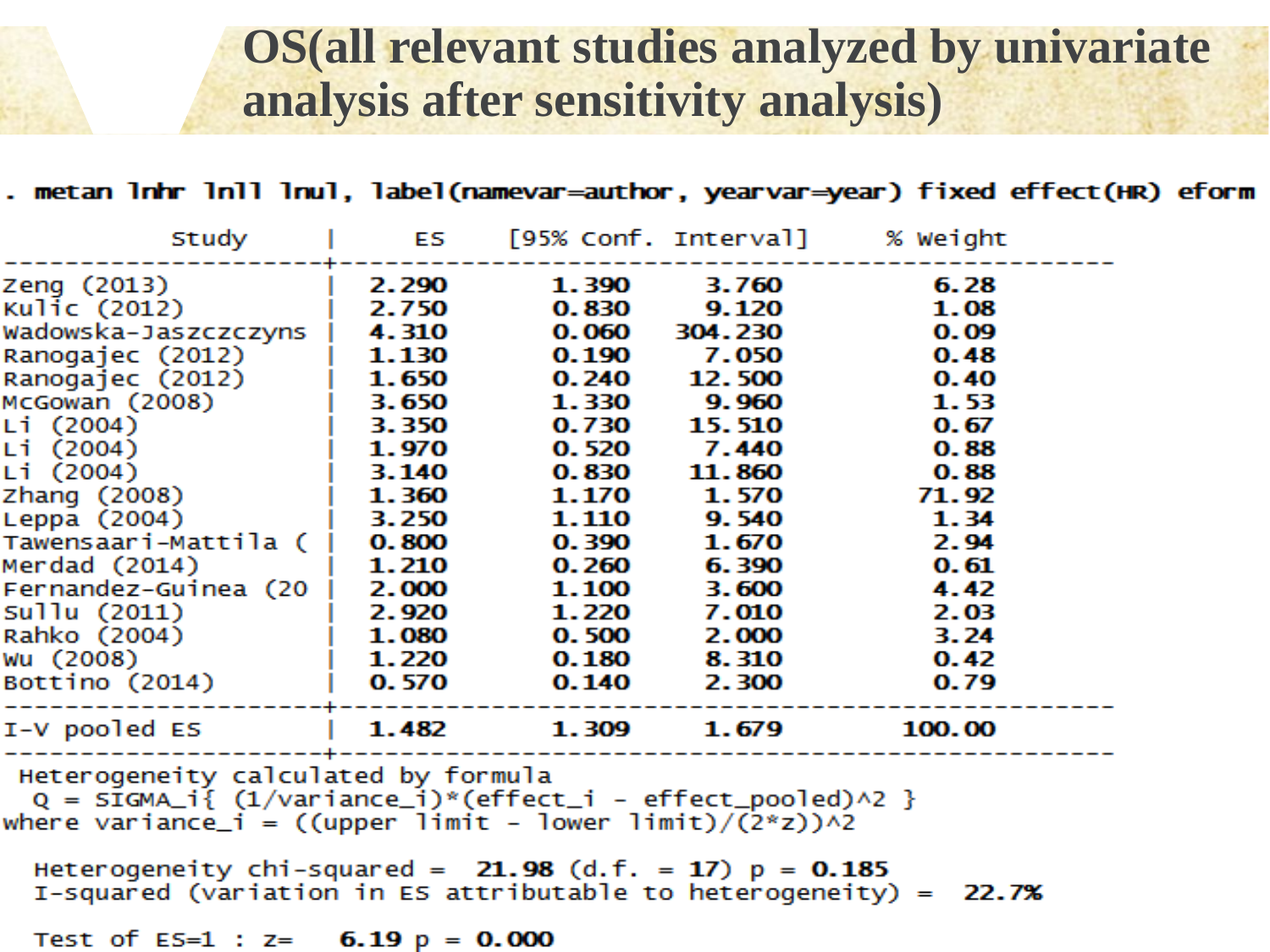

# OS(all relevant studies analyzed by univariate analysis after sensitivity analysis)

## Slide 22
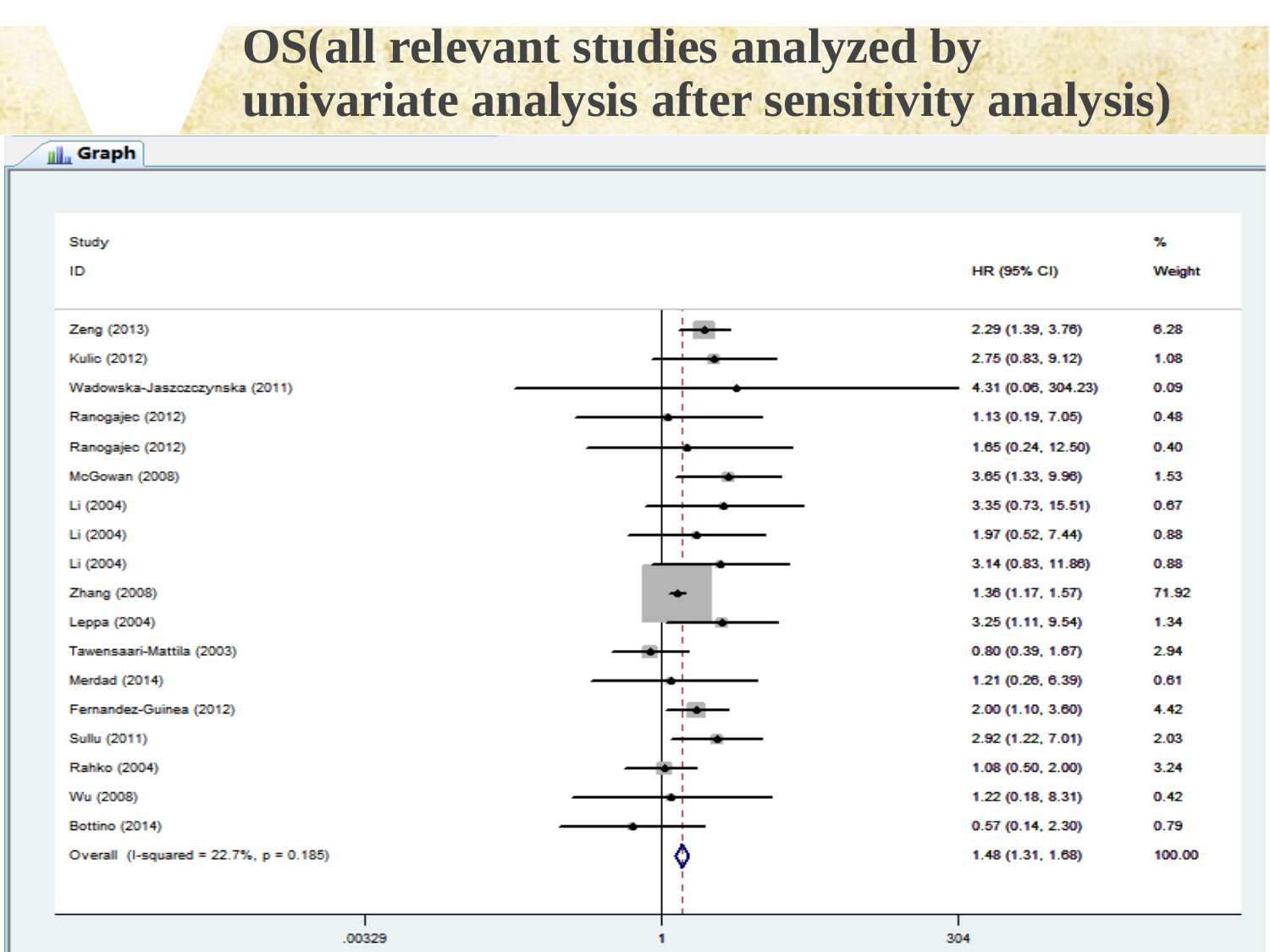

# OS(all relevant studies analyzed by univariate analysis after sensitivity analysis)

## Slide 23
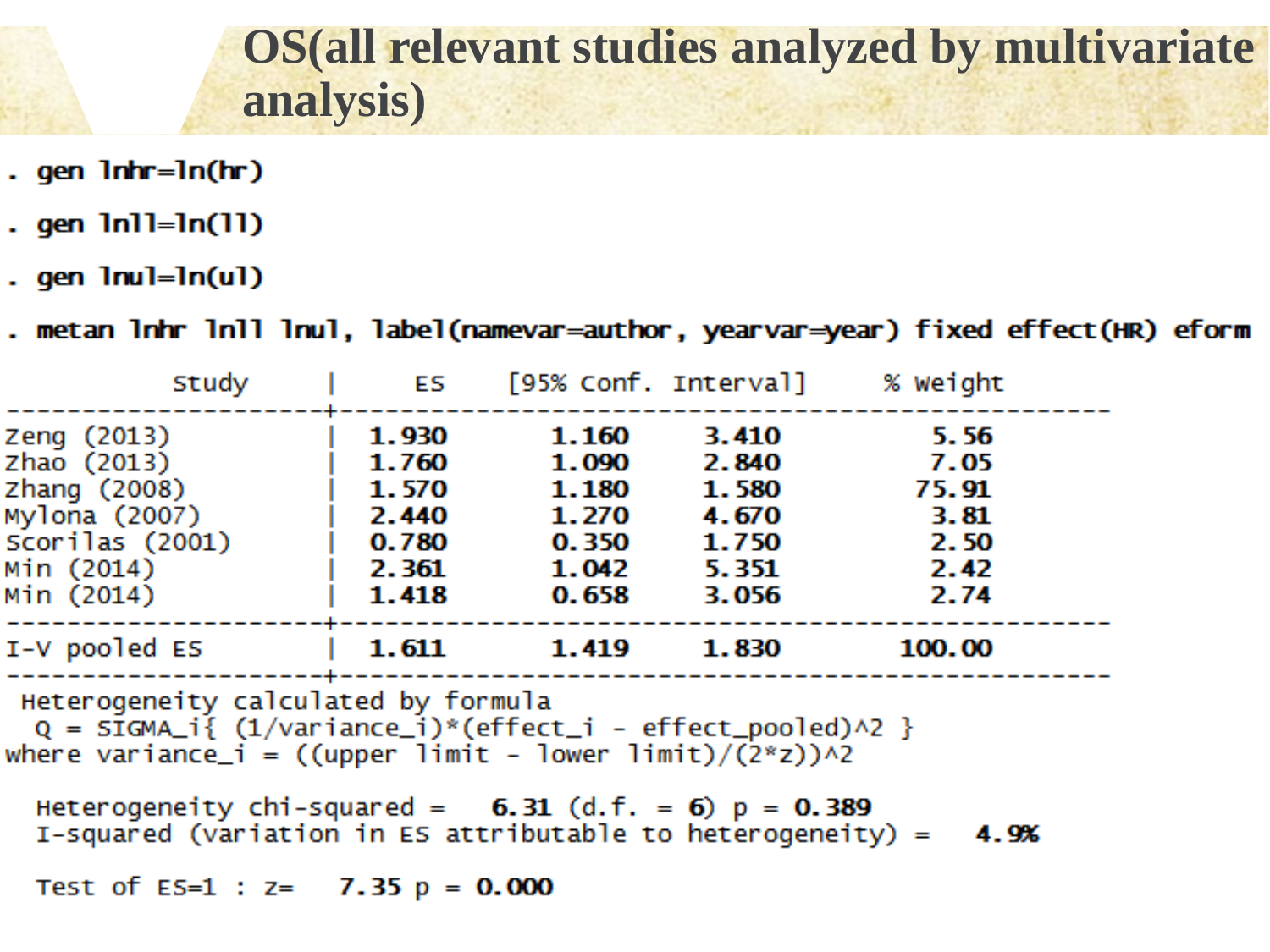

# OS(all relevant studies analyzed by multivariate analysis)

## Slide 24
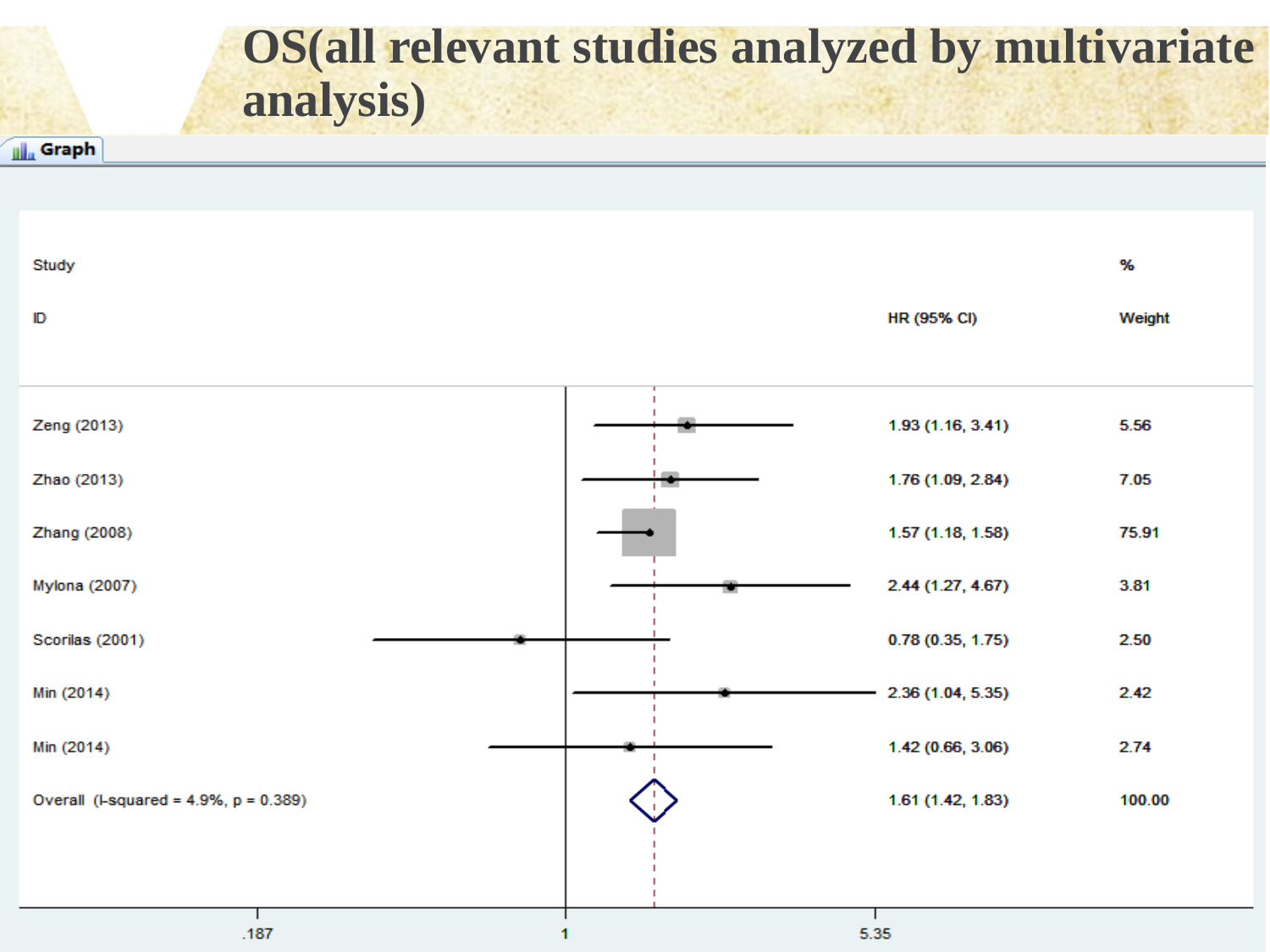

# OS(all relevant studies analyzed by multivariate analysis)

## Slide 25
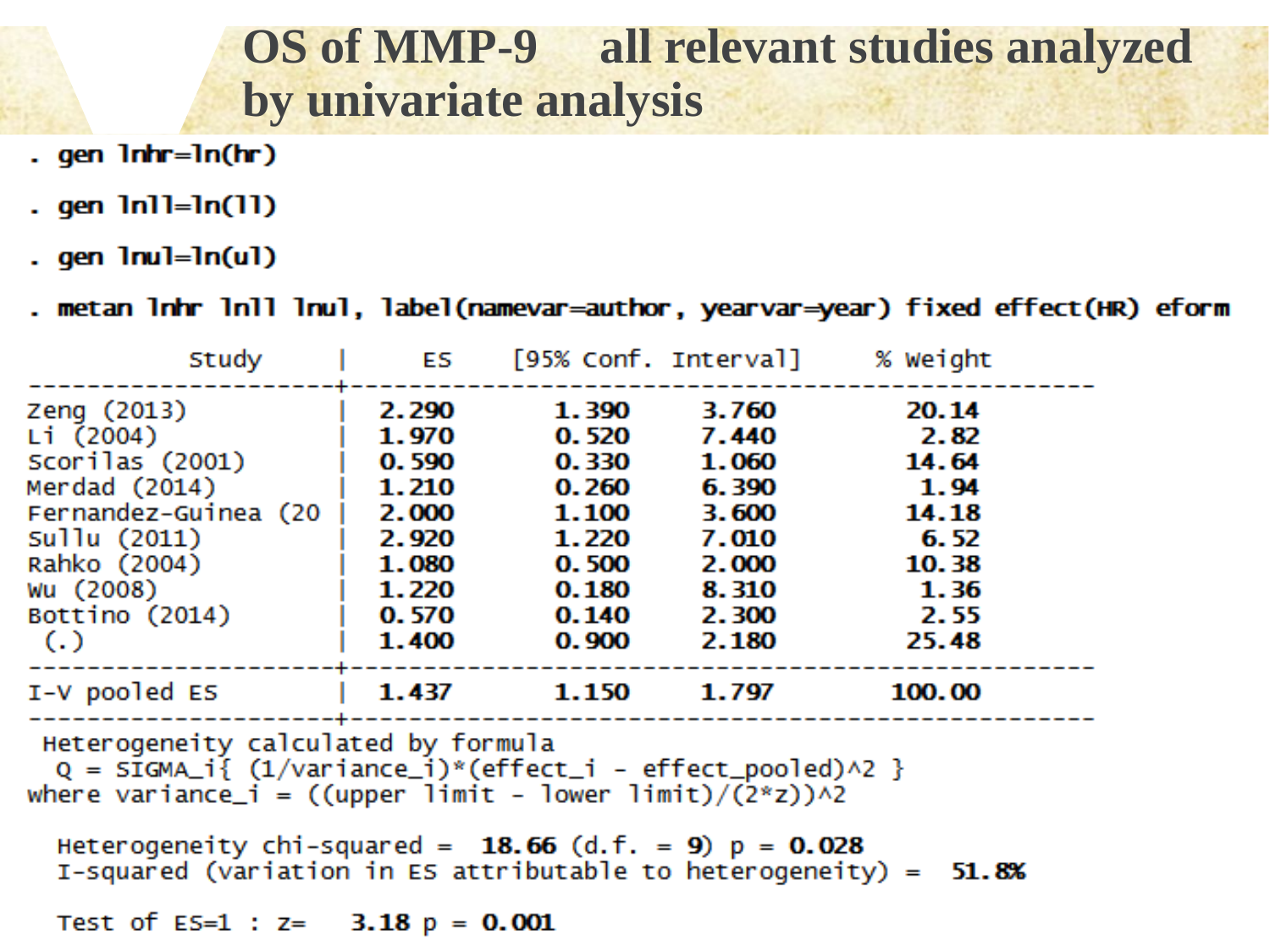

# OS of MMP-9（all relevant studies analyzed by univariate analysis）

## Slide 26
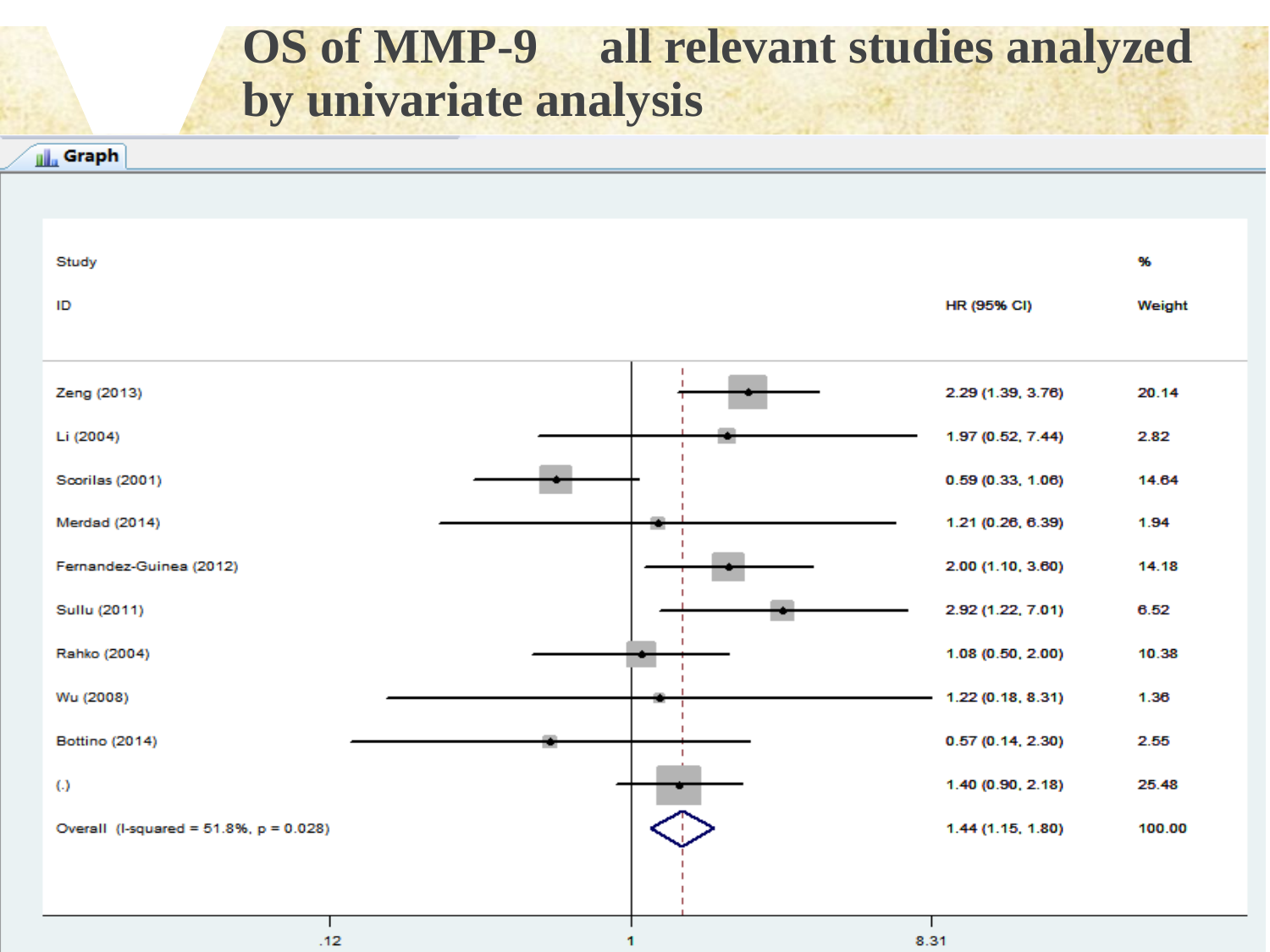

# OS of MMP-9（all relevant studies analyzed by univariate analysis）

## Slide 27
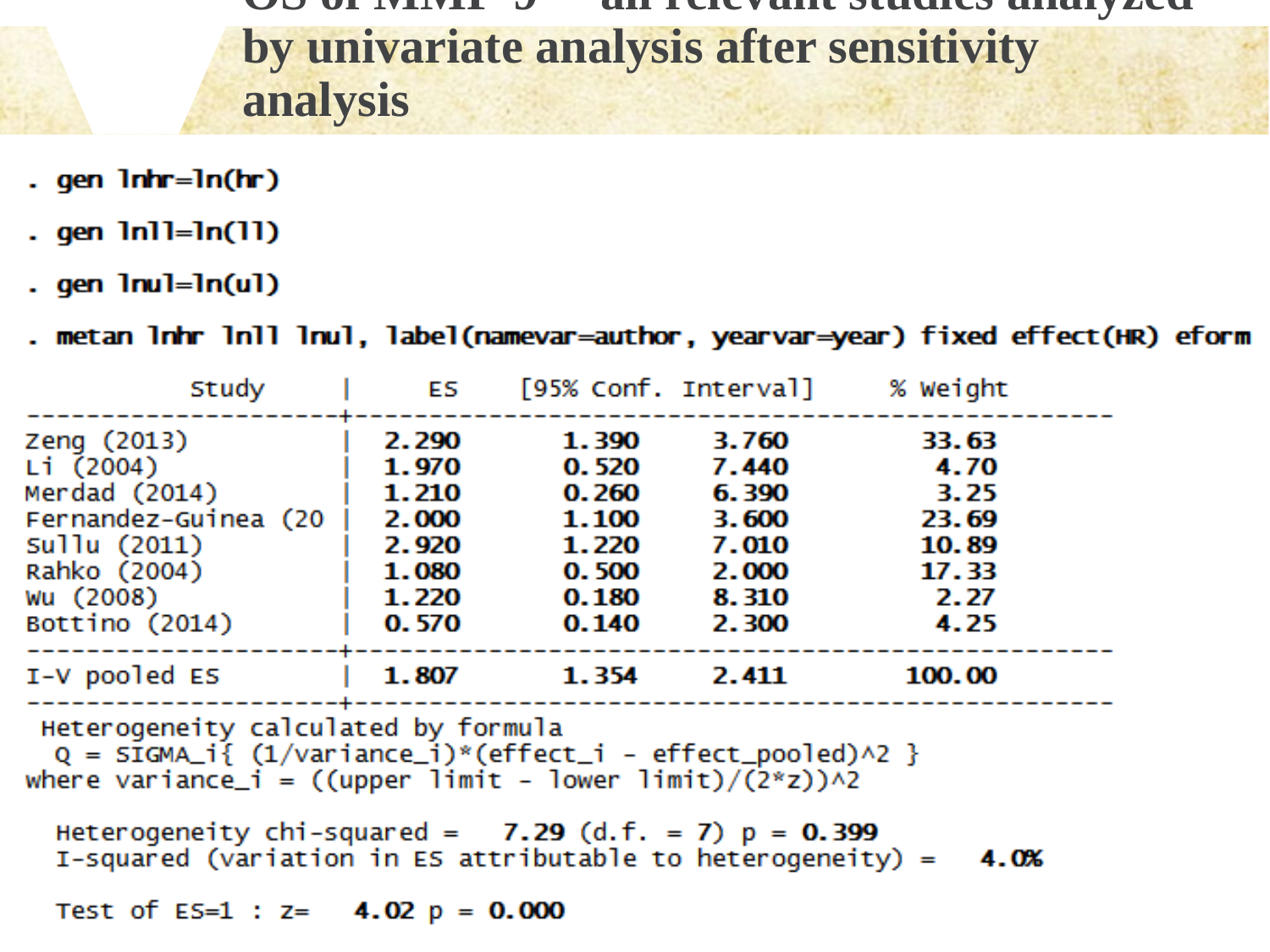

# OS of MMP-9（all relevant studies analyzed by univariate analysis after sensitivity analysis）

## Slide 28
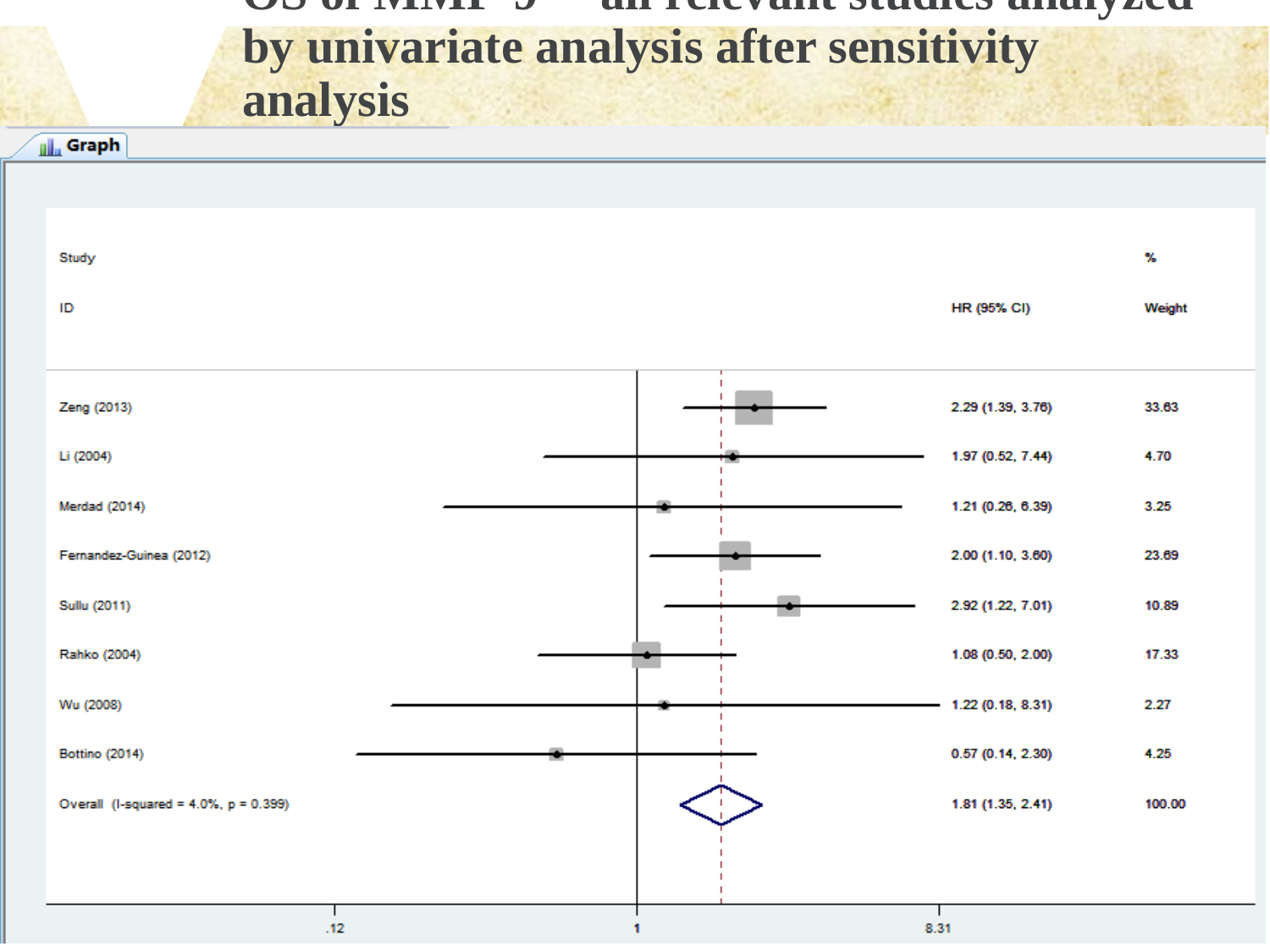

# OS of MMP-9（all relevant studies analyzed by univariate analysis after sensitivity analysis）

## Slide 29
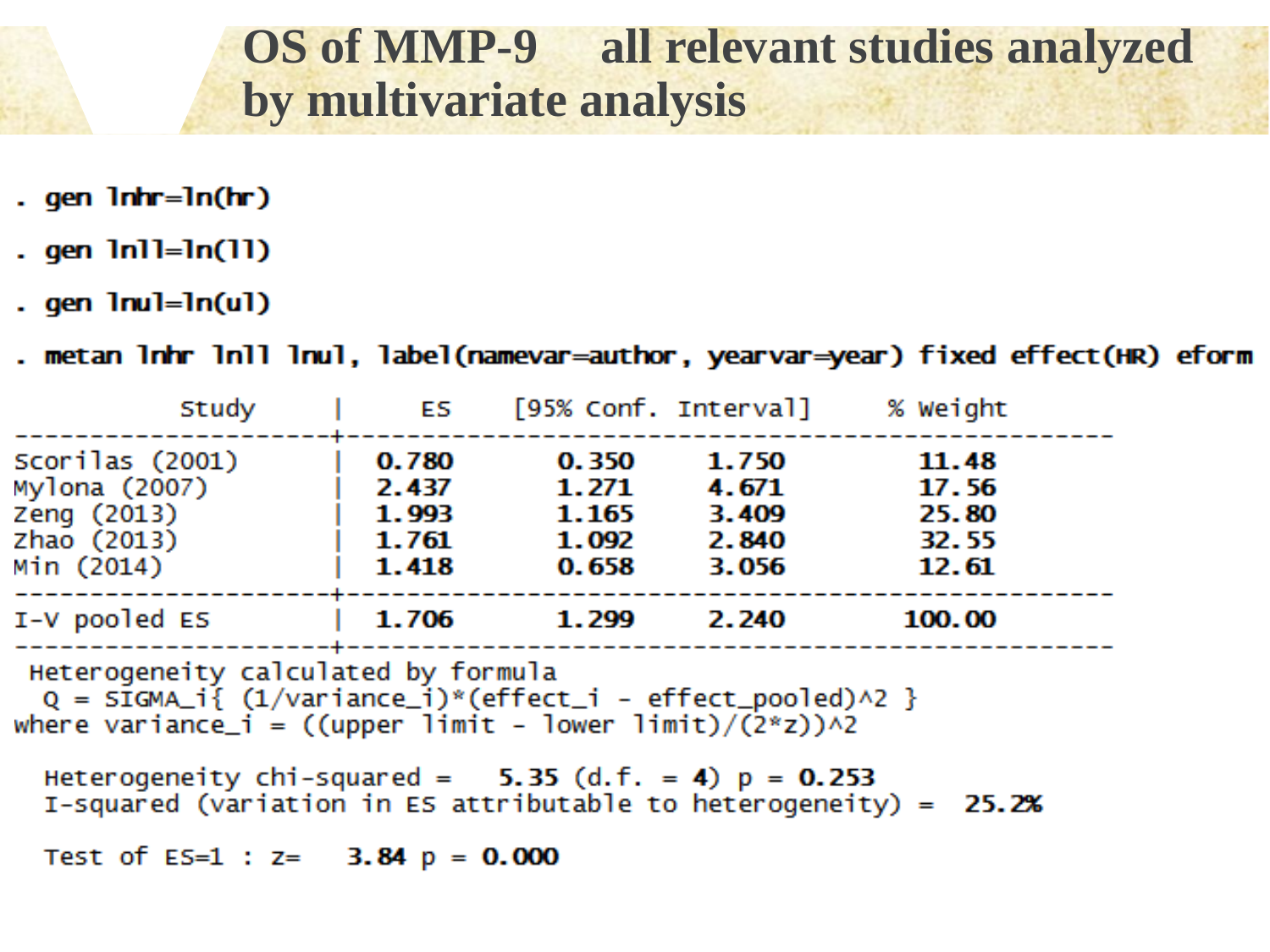

# OS of MMP-9（all relevant studies analyzed by multivariate analysis ）

## Slide 30
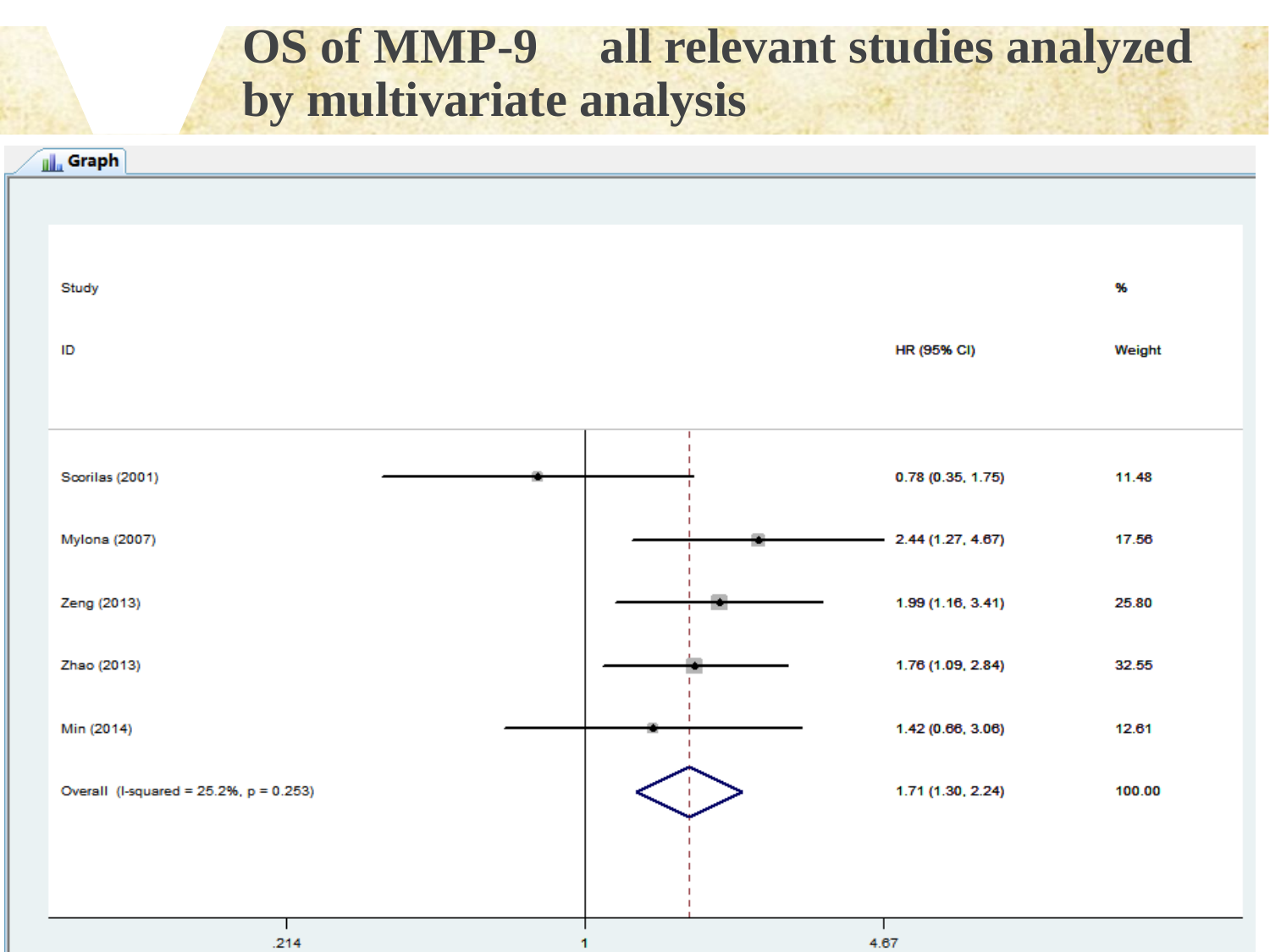

# OS of MMP-9（all relevant studies analyzed by multivariate analysis ）

## Slide 31
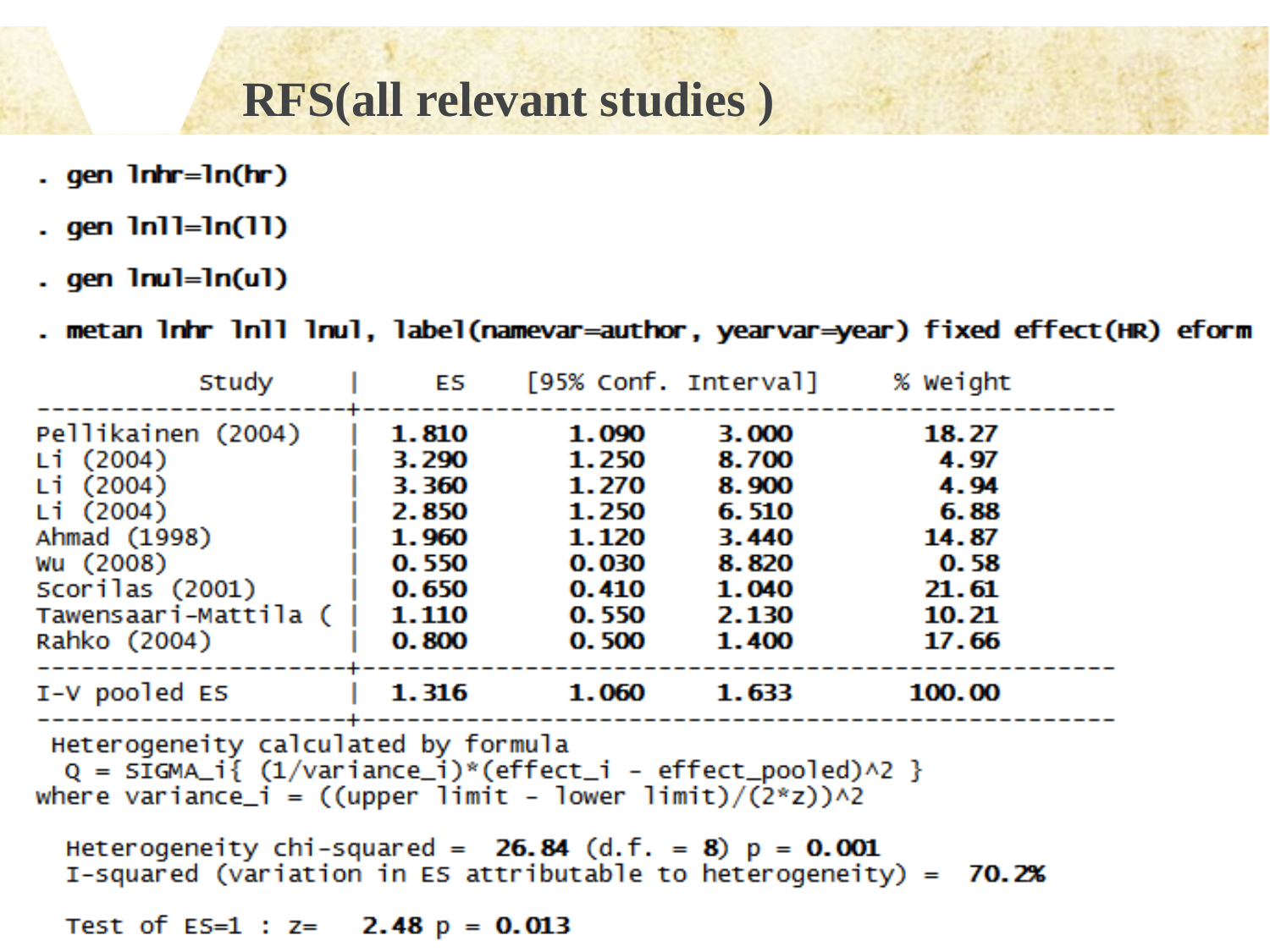

# RFS(all relevant studies )

## Slide 32
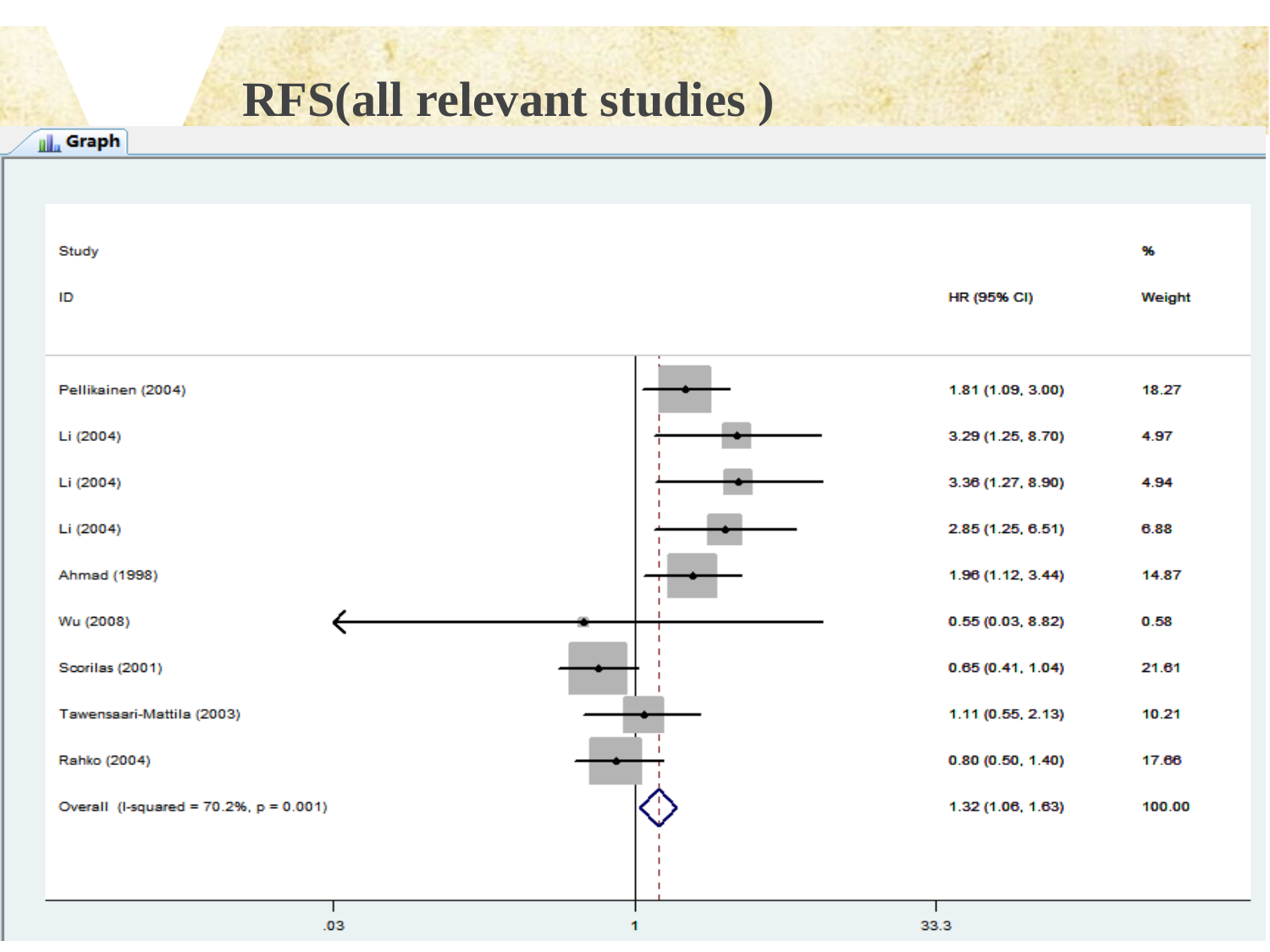

# RFS(all relevant studies )

## Slide 33
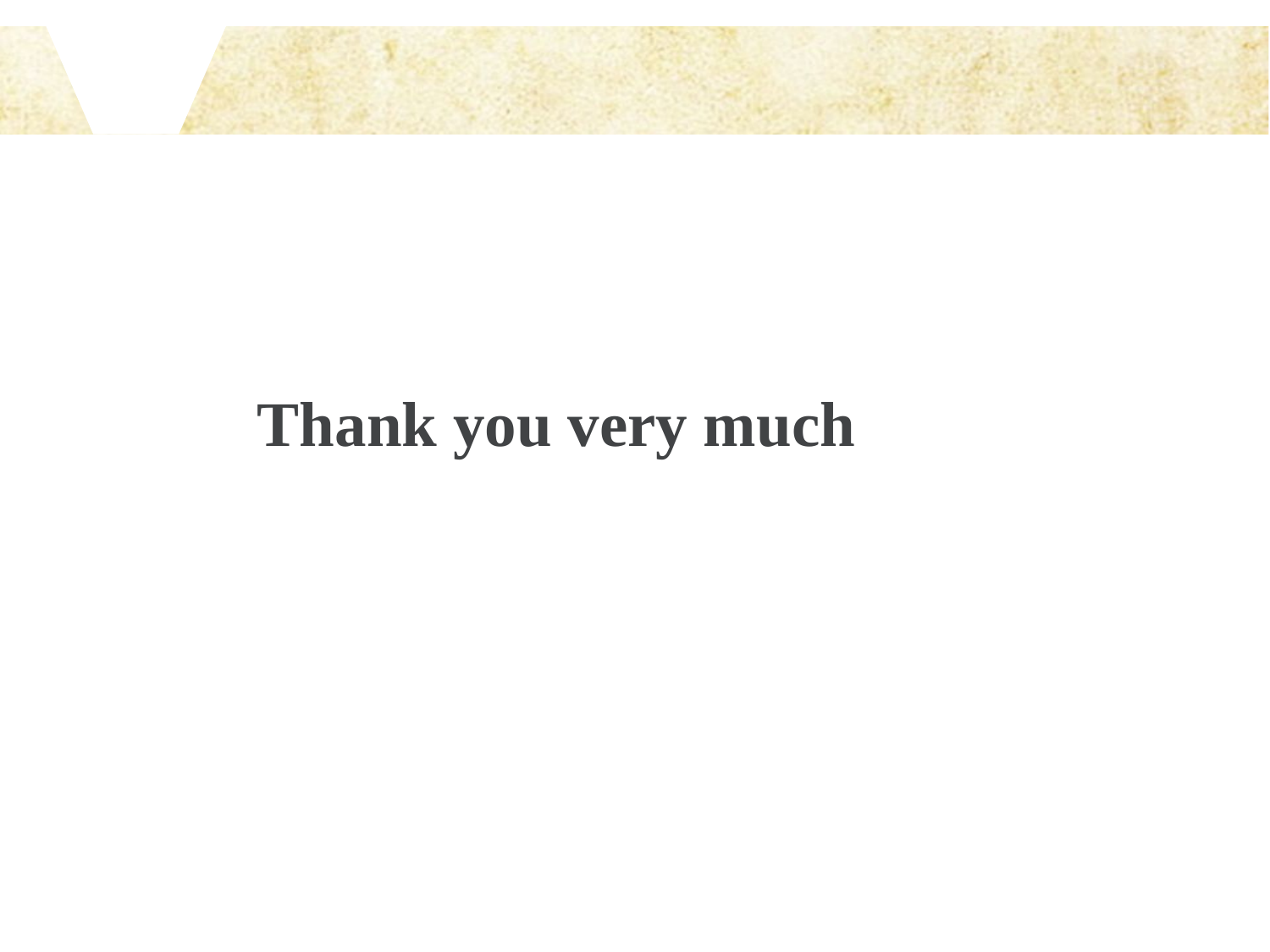

Thank you very much
