# Supplementary material for: Overexpression of MMP Family Members Functions as Prognostic Biomarker for Breast Cancer Patients: A Systematic Review and Meta-Analysis
Source: PLoS One. 2015 Aug 13;10(8):e0135544. doi: 10.1371/journal.pone.0135544 (PMC4535920; doi:10.1371/journal.pone.0135544)
Supplement: S2 File — (DOC) [file pone.0135544.s002.doc]

**No information on prognosis (N=30)**

1. Expression of matrix metalloproteinases in human breast cancer tissues.

(Benson, 2013, PMID: 23568046)

2 .Detection of serum VEGF and MMP-9 levels by Luminex multiplexed assays in patients with breast infiltrative ductal carcinoma.

(Zhang et al. 2014, PMID: 24944618)

3 Expressions of FOXC1 and MMP-7 in molecular subtypes of breast cancer and their association with clinicopathological characteristics

(Cao et al. 2014, PMID: 25187454 2014)

4.Association of Fascin and matrix metalloproteinase-9 expression with poor prognostic parameters in breast carcinoma of Egyptian women.

(Youssef et al. 2014, PMID: 24993803)

5.Expression and correlation of matrix metalloproteinase-9 and heparanase in patients with breast cancer.

(Tang et al. 2014, PMID: 24861922)

6. The level of serum pro-matrix metalloproteinase-2 as a prognostic factor in patients with invasive ductal breast cancer.

(Vucemilo et al. 2014, PMID: 24851607)

7 The prognostic role of interleukin-8 (IL-8) and matrix metalloproteinases -2 and -9 in lymph node-negative untreated breast cancer patients.

(Milovanovic et al. 2013, PMID: 24344010)

8 MT1-MMP in breast cancer: induction of VEGF-C correlates with metastasis and poor prognosis.

(Yao et al. 2013, PMID: 24119788)

9 Matrix metalloproteinase-9 as a potential tumor marker in breast cancer.

(Nanda et al. 2013, PMID: 24099425)

10. CD147, MMP9 expression and clinical significance of basal-like breast cancer.

(Liu et al. 2013, PMID: 23292863)

11 Plasma levels of matrix metalloproteinases 2 and 9 correlate with histological grade in breast cancer patients.

(Vasaturo et al. 2012, PMID: 23255941)

12 Clinical relevance of cyclooxygenase-2 and matrix metalloproteinases (MMP-2 and MT1-MMP) in human breast cancer tissue.

(Mohammad et al. 2012, PMID: 22527932)

13 Matrix metalloproteinases 2, 7, and 9 in tumors and sera of patients with breast cancer.

(Katunina et al. 2011, PMID: 22451887)

14 Role of serum matrix metalloproteinase-2 and -9 to predict breast cancer progression.

(Patel et al. 2011, PMID: 21565179)

15 No evidence indicated that polymorphisms of the matrix metalloproteinases genes were associated with breast cancer risk: an update.

(He et al. 2011, PMID: 21336947)

16 The clinical implications of MMP-11 and CK-20 expression in human breast cancer.

(Cheng et al. 2010, PMID: 19914229)

17 Clinical significance of matrix metalloproteinase 2 and 9 in breast cancer.

(Shah et al. 2009 PMID：19574670)

18 Macrophages in breast cancer: do involution macrophages account for the poor prognosis of pregnancy-associated breast cancer?

(O'Brien et al. 2009, PMID: 19350209)

19 Matrix metalloproteinase-8 functions as a metastasis suppressor through modulation of tumor cell adhesion and invasion.

(Gutiérrez-Fernández et al. 2008, PMID: 18413742)

20 Plasma MMP1 and MMP8 expression in breast cancer: protective role of MMP8 against lymph node metastasis.

(Decock et al. 2008, PMID: 18366705)

21 Expression of nm23, MMP-2, TIMP-2 in breast neoplasm in Zhengzhou Center Hospital, China.

(Peihong et al. 2007, PMID: 17642161)

22 Matrix metalloproteinase expression patterns in luminal A type breast carcinomas.

(Decock et al. 2007, PMID: 17473389)

23 Study of matrix metalloproteinases and their inhibitors in breast cancer.

(Vizoso et al. 2007, PMID: 17342087)

24 The Sample Type used Affects the Levels of Gelatinases (MMP-2 and -9) and their Inhibitors (TIMP-1 and -2) in Circulating Blood of Healthy Controls and Breast Cancer Patients.

(Paula et al. 2007, PMID: 19662197)

25Clinical significance of MT1-MMP mRNA expression in breast cancer.

(Mimori et al. 2001 PMID: 11182063)

26 MMP-9 and MMP-2 gelatinases and TIMP-1 and TIMP-2 inhibitors in breast cancer: correlations with prognostic factors

(Jinga et al. 2006 PMID: 16796815)

27 Metalloproteinases: role in breast carcinogenesis, invasion and metastasis.

(Duffy et al. 2000, PMID: 11250717)

28 Significance of membrane type 1 matrix metalloproteinase expression in breast cancer.

(Ishigaki et al. 1999, PMID: 10391091)

29 MMP-2, MMP-9, VEGF and CA 15.3 in breast cancer.

(Quaranta et al. 2007, PMID: 17972522)

30. Association of Fascin and matrix metalloproteinase-9 expression with poor prognostic parameters in breast carcinoma of Egyptian women.

(Youssef et al. 2014; PMID: 24993803)

**Not written in English (N=10)**

1 Expressions of FOXC1 and MMP-7 in molecular subtypes of breast cancer and their association with clinicopathological characteristics

(Cao et al. 2014, PMID: 25187454)

2 Correlation between expression of metastasis-associated gene 1 and matrix metalloproteinase 9 and invasion and metastasis of breast cancer

(Li et al. 2008, PMID: 19087679)

3 Relationship between the expression of matrix metalloproteinase-13 protein and other biomarkers, prognosis in invasive breast cancer

(Zhang et al. 2008, PMID: 19035119)

4 Clinico-morphological significance of spontaneous necrosis in breast cancer.

(Kriuchkov et al. 2007, PMID: 17663181)

5 Proteases by reactive stromal cells in cancer: an attractive therapeutic target.

(Têtu et al. 2006, PMID: 16980237)

6Expressions of CD147 and matrix metalloproteinase-2 in breast cancer and their correlations to prognosis

(Zhou et al. 2005 PMID: 16004819)

7 Significance of membrane type-1 matrix metalloproteinase expression in breast cancer].

(Yao et al. 2004, PMID: 15566663)

8 Expression of matrix metalloproteinase-9 and its complex in the urine of breast cancer patients].

(Shen et al. 2003, PMID: 14703455)

9 Expression and clinical significance of MMP-2, MMP-9, TIMP-1, and TIMP-2 in breast carcinoma].

(Fan et al. 2003, PMID: 12969531)

10 Expression of matrix metalloproteinase 9 (MMP-9) and laminin-receptor in breast carcinoma and their correlation with tumor metastasis and prognosis].

(Wang et al. 2003 PMID: 12753719)

**No sufficient information to calculate HR and 95%CI (N=5)**

1. High levels of stromelysin-3 correlate with poor prognosis in patients with breast carcinoma.

(Chenard et al. 1996 PMID: 8980245, There was HR but not 95%CI)

2. The level of serum pro-matrix metalloproteinase-2 as a prognostic factor in patients with invasive ductal breast cancer.

(Vucemilo et al. 2014, PMID: 24851607)

**There was K-M survival curve but no number of the corresponding cases to calculate HR and 95%CI (N=3)**

3. Matrix metalloproteinase 26 proteolysis of the NH2-terminal domain of the estrogen receptor beta correlates with the survival of breast cancer patients.

(Savinov et al. 2006, PMID: 16510592)

4. Plasma MMP-9 (92 kDa-MMP) activity is useful in the follow-up and in the assessment of prognosis in breast cancer patients.

(Ranuncolo et al. 2003, PMID: 12866035)

5. Assay of matrix metalloproteinases types 1, 2, 3 and 9 in breast cancer.

(Remacle et al. 1998, PMID: 9528836)

**The method to measure the expression of MMPs didn’t satisfy our inclusions. (N=3)**

**Our inclusion mentioned: (1) MMPs expression was measured in tumor tissue or serum; (2) MMPs protein expression was measured instead of mRNA**

1 High MMP-1 mRNA expression is a risk factor for disease-free and overall survivals in patients with invasive breast carcinoma.

(Cheng et al. 2008, PMID: 17663001, measured mRNA)

2 The value of MMP-9 for breast and non-small cell lung cancer patients' survival.

(Schveigert et al.2013, PMID, 23640949, measured in blood)

3 Correlation between stromelysin-3 mRNA level and outcome of human breast cancer.

(Engel et al. 1994, PMID: 7927875, measured mRNA)

**The combination of MMPs and other biomarkers was evaluated to predict the prognosis of breast cancer not the independent expression of MMPs (N=3)**

1. Prognostic values of ETS-1, MMP-2 and MMP-9 expression and co-expression in breast cancer patients

([Puzovic](http://www.ncbi.nlm.nih.gov/pubmed/?term=Puzovic V%5BAuthor%5D&cauthor=true&cauthor_uid=24645837), 2014; PMID: 24645837)

2. Plasma levels of the MMP-9: TIMP-1 complex as prognostic biomarker in breast cancer: a retrospective study

([Thorsen](http://www.ncbi.nlm.nih.gov/pubmed/?term=Thorsen SB%5BAuthor%5D&cauthor=true&cauthor_uid=24330623) 2013; PMID: 24330623)

3. Prognostic significance of the combined expression of matrix metalloproteinase-9, urokinase type plasminogen activator and its receptor in breast cancer as measured by Northern blot analysis.

(Pacheco et al. 2001 PMID: 11288958)

**Review (N=1)**

1. Candidate prognostic markers in breast cancer: focus on extracellular proteases and their inhibitors.

(Roy et al. 2014 PMID: 25114586)
